# Discovery and binding mode of small molecule inhibitors of the apo form of human TDO2

**DOI:** 10.1038/s41598-024-78981-4

**Published:** 2024-11-14

**Authors:** Carina Lotz-Jenne, Roland Lange, Sylvaine Cren, Geoffroy Bourquin, Laksmei Goglia, Thierry Kimmerlin, Micha Wicki, Manon Müller, Nadia Artico, Sabine Ackerknecht, Philippe Pfaff, Christoph Joesch, Aengus Mac Sweeney

**Affiliations:** Drug discovery, Idorsia Pharmaceuticals Limited, Hegenheimermattweg 91, Allschwil, Basel-Land, 4123 Switzerland

**Keywords:** Enzymes, Proteins, Structural biology, Small molecules, Oxidoreductases, Tumour immunology

## Abstract

Tryptophan-2,3-dioxygenase (TDO2) and indoleamine-2,3-dioxygenase (IDO1) are structurally distinct heme enzymes that catalyze the conversion of L-tryptophan to N-formyl-kynurenine, and play important roles in metabolism, inflammation, and tumor immune surveillance. The enzymes can adopt an inactive, heme-free (apo) state or an active, heme-containing (holo) state, with the balance between them varying dynamically according to biological conditions. Inhibitors of holo-TDO2 are known but, despite several advantages of the heme-free state as a drug target, no inhibitors of apo-TDO2 have been reported. We describe the discovery of the first apo-TDO2 binding inhibitors, to our knowledge, and their inhibition of cellular TDO2 activity at low nanomolar concentrations. The crystal structure of a potent, small molecule inhibitor bound to apo-TDO2 reveals its detailed binding interactions within the large, hydrophobic heme binding pocket of the active site.

**Supplementary Information:**

The online version contains supplementary material available at 10.1038/s41598-024-78981-4.

## Introduction

Tryptophan-2,3-dioxygenase (TDO2) and indoleamine-2,3-dioxygenase 1 (IDO1) catalyze the same reaction: the conversion of L-tryptophan (Trp) to N-formyl-kynurenine (NFK). Both enzymes evolved to bind heme for substrate binding and catalysis but are structurally distinct: IDO1 is monomeric, while TDO2 is tetrameric with two independent TDO2 molecules contributing to each of its four active sites. Together, they play important roles in metabolism, inflammation, and tumor immune surveillance. Their cellular enzymatic activity levels depend on the relative proportion of apo (inactive, heme-free) and holo (active, heme containing) forms, which vary dynamically according to diverse biological signals including nitric oxide (NO) levels^1–3^. Both IDO1 and TDO2 exist, most likely predominantly, in their heme-deficient forms in cells and tissues. Studies from approximately fifty years ago showed low (25–50%) heme saturation in healthy rat liver TDO2^4–6^, increasing to 80–100% saturation within 2 hours after giving the animals Trp^7,8^ or hemin^5,6^. The enzyme GAPDH delivers heme to apo-TDO2, potentially via a direct protein-protein interaction. Inhibition of the GAPDH interaction with apo-TDO2 has been proposed as a potential strategy to inhibit the formation of holo-TDO2^2^. Since the enzymatic activity of IDO1 and TDO2 have been shown to contribute to the immune-suppressive condition in the tumor microenvironment (reviewed in^9^), small molecule inhibitors are regarded as a therapeutic option to reinvigorate anti-tumor immunity. Small molecule, active-site inhibitors binding to apo-IDO1^10,11^, holo-IDO1 ^12–14^ and holo-TDO2^15^ have been reported, as well as dual inhibitors of holo-IDO1 and holo-TDO2 ^16–18^. Despite the extensive research in this field, and the likely benefits of targeting the apo form of the enzyme, no inhibitors of apo-TDO2 have been reported to date.

Several IDO1 selective inhibitors have been tested in oncology clinical trials, often in combination with immune checkpoint inhibitors, but have failed to demonstrate sufficient efficacy to date^19^. Of the dual holo-IDO1/TDO2 inhibitors that entered phase I clinical trials, results were reported only for M4112 (Merck KGaA, Darmstadt, Germany), which was discontinued due to an insufficient pharmacodynamic effect^20^. Most of the clinical studies in the IDO1 field, including the only completed phase three study, have been performed with the holo-IDO1 inhibitor epacadostat. In the phase three clinical trial, epacadostat failed to show improved progression-free survival or overall survival compared with placebo in patients with unresectable or metastatic melanoma (all patients additionally received the immune checkpoint inhibitor pembrolizumab)^21^. While many hypotheses have been raised as to why the trial was unsuccessful, it is likely that the epacadostat dose administered was insufficient to block the enzymatic activity of IDO1 ^22^.

For IDO1, the apo form of the enzyme appears to be more suitable as a drug target than the holo form. In particular, BMS-986,205 (linrodostat) shows higher potency in cell-based assays and in vivo pharmacodynamic studies than inhibitors of the holo form^22^. Apo-binding inhibitors circumvented competition of the Trp substrate with the inhibitor and in addition led to sustained IDO1 inhibition due to delayed rebinding of heme to the apoenzyme following inhibitor dissociation^10,23^. Despite the high likelihood that the same potential therapeutic benefits apply to apo-TDO2 as to apo-IDO1, no inhibitors targeting apo-TDO2 have been reported to date. Therefore, we screened a diverse compound library and identified the first inhibitors of apo-TDO2 to our knowledge. We believe that the discovery and characterization of these small molecules paves the way for a new class of TDO2 inhibitors with a different mechanism of action and the potential for greater in vivo efficacy. While apo-TDO2 is catalytically inactive, the term “apo-TDO2 inhibitor” is used to describe compounds that bind to apo-TDO2, compete with heme binding and thus prevent the formation of enzymatically active holo-TDO2.

## Results

### Identification of the inhibitor Cpd-1 in a cellular TDO2 activity assay

**Fig. 1:**
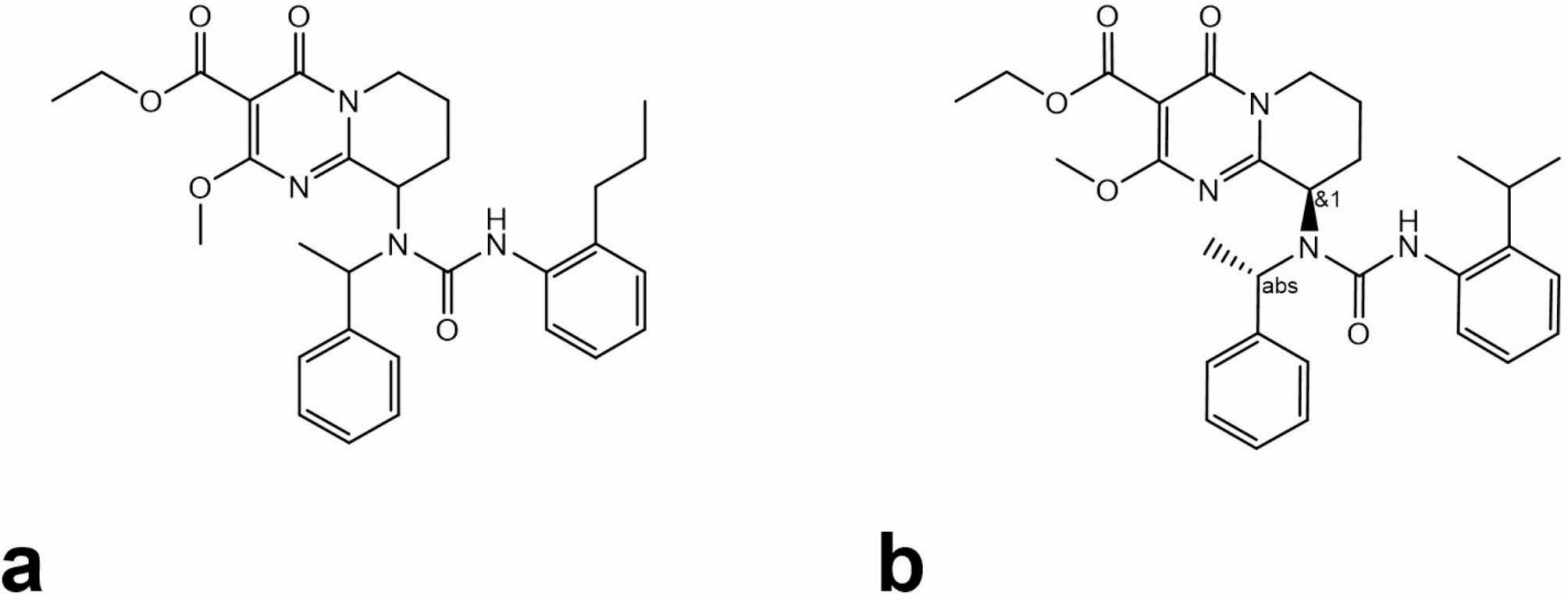
The chemical structures of the initial hit Cpd-1 **(A)** and its more potent close analog Cpd-2 **(B)**. Cpd-1 is a mixture of four stereoisomers and Cpd-2 is a mixture of two stereoisomers, of which only the stereoisomer drawn is expected to bind to apo-TDO2, based on the crystal structure of Cpd-2 bound to apo-TDO2.

The inhibitor compound 1 (Cpd-1) (IUPAC name ethyl 9-(3-(2-propylphenyl)-1-(1-phenylethyl)ureido)-2-methoxy-4-oxo-6,7,8,9-tetrahydro-4*H*-pyrido[1,2-*a*]pyrimidine-3-carboxylate) was identified as a hit in a high throughput screening campaign of a diverse set of 244,625 compounds in a human TDO2 cellular assay utilizing SW48 cells, which express a high level of endogenous TDO2. TDO2 activity was assessed by measuring the accumulation of the TDO2 product NFK and downstream product KYN in the supernatant^15^. The potent inhibitor compound 2 (Cpd-2) (IUPAC name ethyl 9-(3-(2-isopropylphenyl)-1-((*S*)-1-phenylethyl)ureido)-2-methoxy-4-oxo-6,7,8,9-tetrahydro-4*H*-pyrido[1,2-*a*]pyrimidine-3-carboxylate) was identified during initial structure activity relationship studies of Cpd-1 analogs. The chemical structures of both inhibitors are shown in Fig. 1. Cpd-1 and Cpd-2 displayed IC_50_ values of 327 nM and 14.8 nM, respectively, in the TDO2 cellular assay. While Cpd-1 is a mixture of four diastereomers, Cpd-2 is a mix of the two diastereomers shown in Fig. 1B. Interference of the compounds with the TDO2 cellular assay via any cytotoxic effect was excluded using an SW48 cell viability assay, with IC_50_ values of greater than 10 µM for both compounds.

In keeping with its apo-TDO2 binding mode of action, Cpd-1 displayed only weak inhibition of the holo form of TDO2 in a standard 30 min enzymatic assay at room temperature following 30 min pre-incubation of the compound with holo-TDO2 (enzymatic assay IC_50_ of 17.4 µM, 53-fold less potent than in the cellar assay). The IC_50_ curve is shown in supplementary Fig. S1 online. We speculate that the observed IC_50_ shift may be due to the kinetics of heme dissociation from holo-TDO2 and/or the absence of alternative heme binding proteins in the assay system. The structurally similar Cpd-2 was not tested in the enzymatic assay.

### Measuring IDO1 inhibition

A cellular assay similar to the TDO2 assay was used to measure IDO1 inhibition in SKOV3 cells. Cpd-1 and Cpd-2 showed almost no inhibition of IDO1, with respective IC_50_ values of 5.7 µM and > 10 µM. IC_50_ curves are shown in Fig. S4 online.

### Differential scanning fluorimetry (DSF)

As a further proof of binding to apo-TDO2, differential scanning fluorimetry (DSF) was used to measure the thermal stabilization of apo-TDO2 by Cpd-1 and Cpd-2 in the presence of the known exosite binding compound α-methyltryptophan (AMT) at 500 µM concentration (Table S2 and Fig. S5 online). At a concentration of 25 µM, Cpd-1 and Cpd-2 increased the melting temperature of apo-TDO2 by 11.2 and 14.5 °C, respectively, compared to a melting temperature of 59.2 °C in the absence of inhibitor.

An exosite that binds both the substrate Trp and its α-methyl analog AMT has been identified previously in holo-TDO2^24^. Biochemical and cellular analyses indicated that AMT binding to the exosite of holo-TDO2, while not affecting the catalytic properties, prevented its proteolytic degradation by the ubiquitin-dependent proteasomal pathway and stabilized the isolated holo-TDO2 enzyme against heat, urea and proteases^24^.

We observed high initial fluorescence values (below the apo-TDO2 melting temperature) in the absence of an apo-TDO2 inhibitor, which we speculate is due to binding of the Sypro orange dye in the unoccupied, hydrophobic heme binding pocket. AMT led to an improved apo-TDO2 melting curve quality (Fig. S5 online) and was therefore included in all apo-TDO2 inhibitor DSF measurements.

### Monitoring heme-competition by holo-TDO2 UV-VIS absorbance

**Fig. 2:**
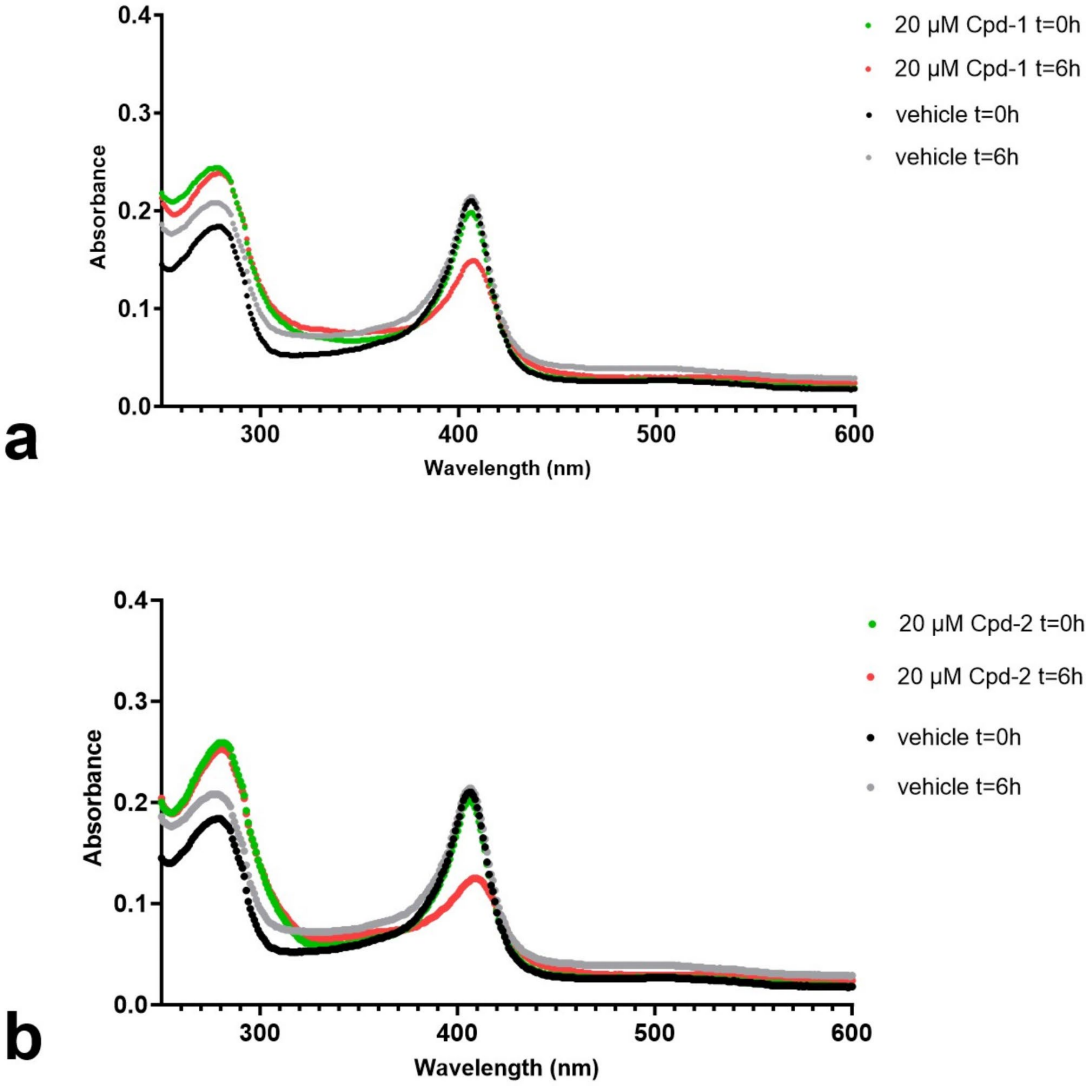
The UV-VIS absorption spectrum of holo-TDO2 in the presence of Cpd-1 **(A)** or Cpd-2 **(B)** or the vehicle control. The incubation of 5 µM holo-TDO2 with 20 µM inhibitor shows a reduction of the absorbance peak at 405 nm (the Soret band) after 6 h of coincubation at 37 °C, indicating heme loss due to competitive binding of Cpd-1 or Cpd-2 (red dotted line). The increase of absorbance at 280 nm of holo-TDO2 in the presence of Cpd-1 or Cpd-2 compared to the vehicle control is likely due to compound absorbance at this wavelength.

To explore whether the identified apo-binders could compete with heme-binding to TDO2, a UV-visible absorption spectrum of holo-TDO2 was measured in the absence or presence of compounds. Heme-containing proteins exhibit a characteristic absorbance peak at 405 nm, known as the Soret band, which corresponds to the electronic state of the heme. A shifted Soret peak can indicate a heme protein-ligand interaction, and a reduced intensity indicates loss of protein bound heme (although free heme also absorbs with a peak at approximately 400 nm, it is very poorly soluble). The holo-TDO2 absorption spectrum in the presence of Cpd-1 or Cpd-2 showed a clear time-dependent reduction in the Soret band intensity, similar to that reported for known IDO1 apo-binders^23^, while no reduction was seen in the presence of the vehicle control (Fig. 2).

### Structure of Cpd-2 bound to apo-TDO2

To further validate its binding to apo-TDO2, and to determine the inhibitor binding site and interactions, the X-ray crystal structure of apo-TDO2 inhibitor in complex with the more potent inhibitor Cpd-2 was determined. Two steps were essential for successful co-crystallization of apo-TDO. First, apo-TDO2 was expressed using an *E. coli* strain that is unable to synthesize heme. Second, the exosite binder AMT was added to apo-TDO2 because of its stabilizing effect as observed in DSF experiments. In the presence of AMT, apo-TDO2 could be crystallized both alone and in complex with the inhibitor Cpd-2.

The 2.61 Å resolution structure of Cpd-2 in complex with apo-TDO2 revealed the inhibitor bound to the active site (including the heme binding pocket) of all four TDO2 molecules of the homo-tetramer. Cpd-2 is a mixture of two diastereomers, of which only ethyl (*R*)-9-(3-(2-isopropylphenyl)-1-((*S*)-1-phenylethyl)ureido)-2-methoxy-4-oxo-6,7,8,9-tetrahydro-4*H*-pyrido[1,2-*a*]pyrimidine-3-carboxylate (as depicted in Fig. 1) was observed in the co-crystal structure. Omit electron density maps for the four independent copies of the inhibitor are shown in Supplementary Fig. S2 online.

The real space correlation coefficients (2Fo-Fc) of the fully refined diastereomers were calculated using the “buster-report” software (Global Phasing) for both possible stereoisomers ((R, S) and (S, S)). The correlation coefficient of the (R, S) stereoisomer was marginally higher than (S, S) in each of the four TDO2 molecules, with an average value of 0.932 for (R, S) and 0.923 for (S, S). No geometry flags were reported for the (R, S) stereoisomer. By contrast, unusual dihedrals for the amide adjacent to the stereocenter were reported for the (S, S) stereoisomer after refinement, indicating that significantly more distortion was required to fit the (S, S) stereoisomer to the electron density. The distortion was observed for all four (S, S) stereoisomers in the TDO2 homotetramer.

**Fig. 3:**
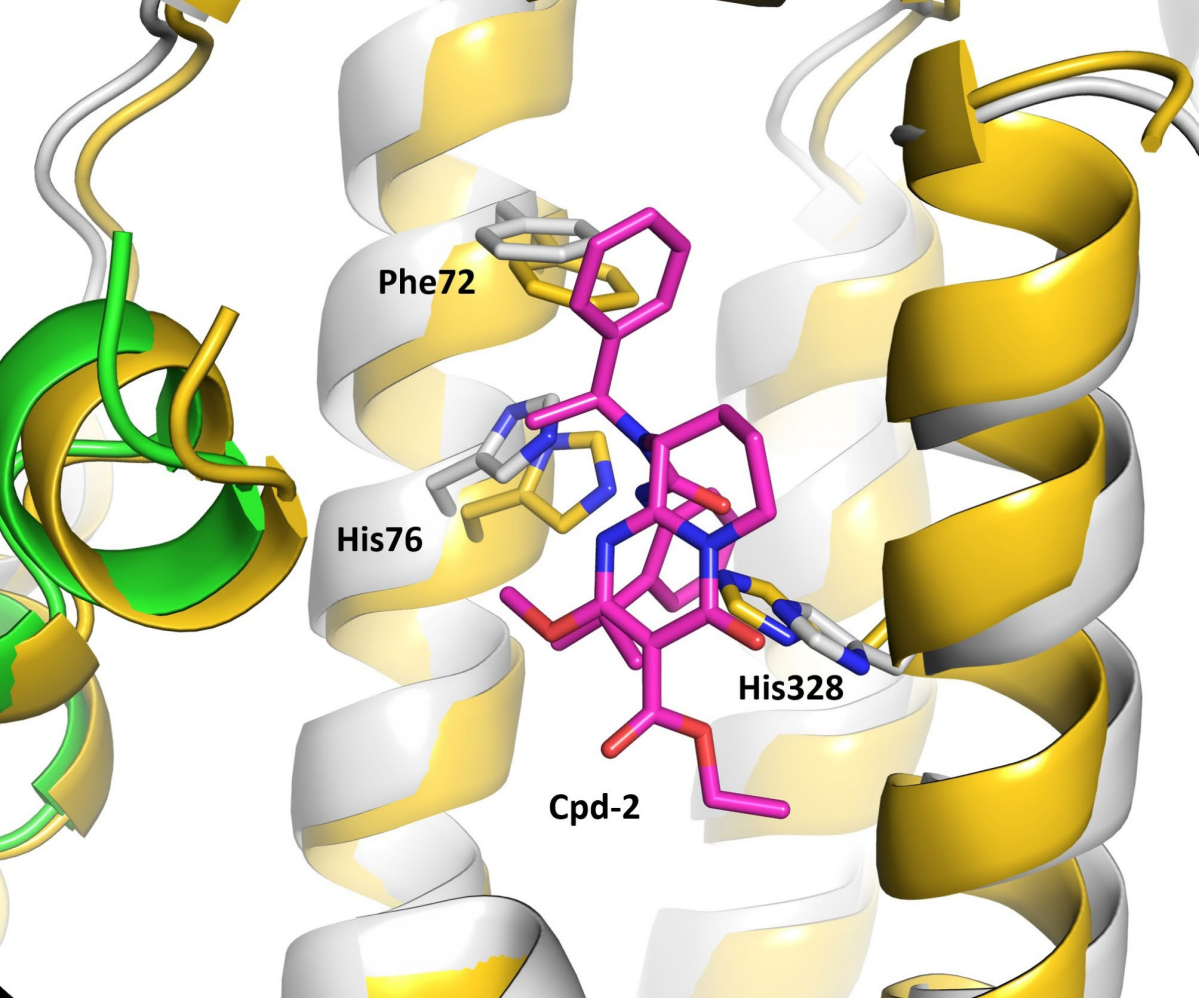
Superposition of the apo-TDO2 Cpd-2 complex with the published apo-TDO2 structure. The apo-TDO2 Cpd-2 complex is shown with protein in green and grey, and inhibitor in magenta, and the published apo-TDO2 structure (PDB entry ID 4PW8) is shown in gold. Each TDO2 active site is formed by two monomers of the homotetramer, shown here in grey and green for the apo-TDO2 Cpd-2 complex. In stick format, atoms are colored blue (N) and red (O). Loop 148–156 is omitted for clarity.

Significantly, the overall conformation of apo-TDO2 in the inhibitor complex resembles that of the published 2.90 Å structure of apo-TDO2 alone (PDB ID 4PW8)^25^, except for an “induced fit” widening of the binding pocket in the inhibitor complex (Fig. 3). The r.m.s.d. of the ordered parts of chain A of both structures (residues 41 to 382) is 1.38 Å. Residues Phe72, His76 and His328 are shifted away from the inhibitor by 1.0-1.5 Å, together with the neighboring residues of the associated alpha-helices. The shortest distance between the His76 and His328 side chain atoms is 4.0 Å in apo-TDO2 and 6.9 Å in the Cpd-2 / apo-TDO2 complex. This first apo-TDO2 structure was published in 2014, several years before the discovery of apo forms of IDO1 and TDO2 in vivo, and its potential physiological relevance was therefore not discussed.

**Fig. 4:**
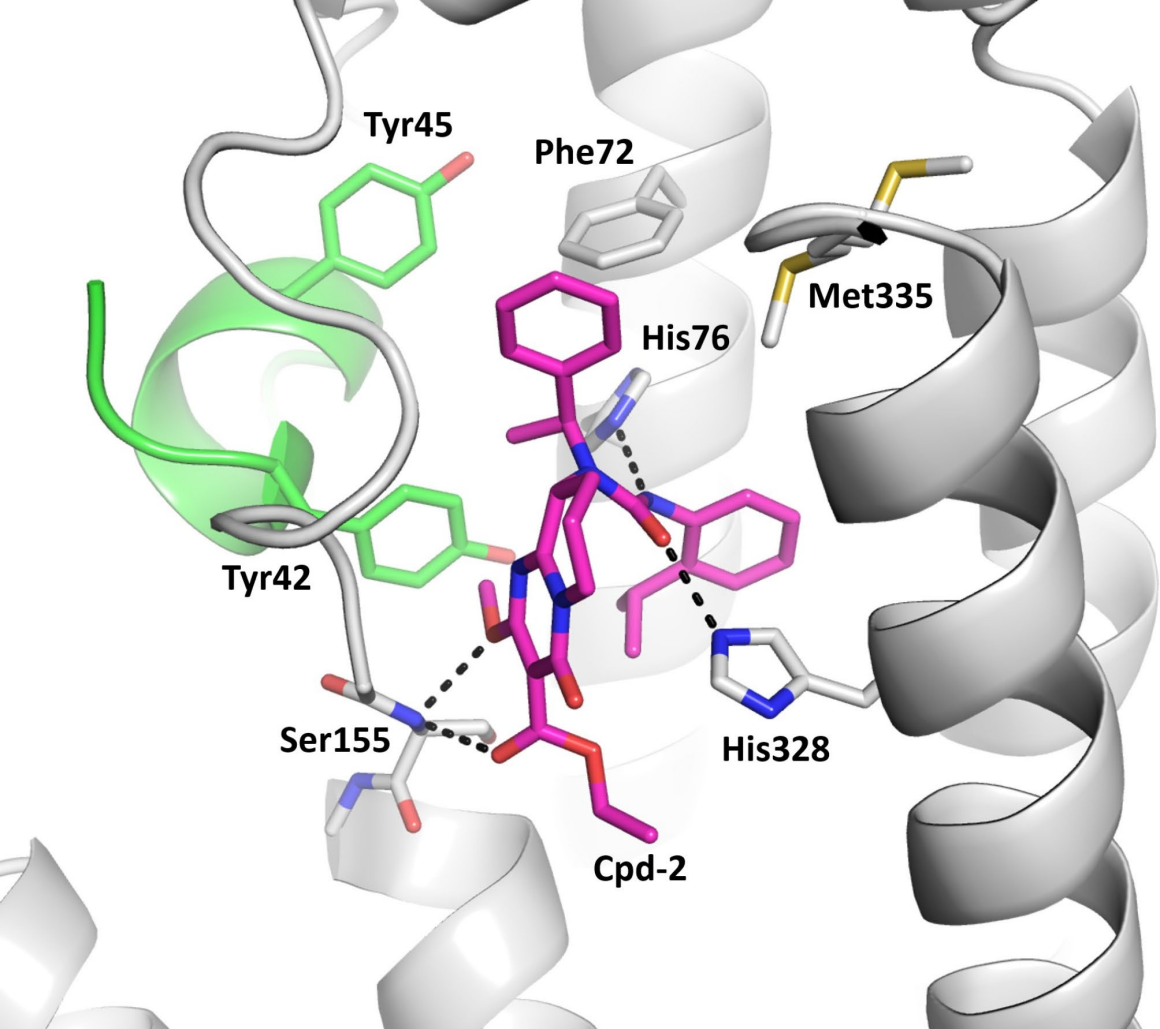
Binding interactions of the inhibitor Cpd-2 in the heme binding pocket of apo-TDO. Each TDO2 active site is formed by two monomers of the homotetramer, shown here in grey and green. Hydrogen bond interactions between the inhibitor and TDO2 are shown as black dashes. In stick format, atoms are colored blue (N), red (O) and yellow (S). Cpd-2 is shown in magenta.

Cpd-2 occupies both the heme and substrate binding pockets of the active site and, in keeping with its potent inhibition of TDO2 activity in the cellular assay, forms multiple binding interactions with TDO2 (Fig. 4). Both benzyl rings of the inhibitor are bound in hydrophobic pockets formed by phenylalanine, leucine and tryptophan side chains. The benzyl rings are bridged by a central urea linker that forms hydrogen bonds with the side chains of His328 and His76 (both are key residues for holo-TDO2 activity, with His328 anchoring the heme via its iron atom and His76 binding the substrate Trp). The backbone nitrogen of Ser155 forms an additional bifurcated hydrogen bond interaction with the inhibitor. The larger fused ring substituent of the inhibitor binds between loop 145–155 and His328 and may serve to stabilize the loop conformation and shield the hydrogen bond between the inhibitor and His328 from competing water molecules.

Ignoring the potential role of cellular permeability differences between the two compounds, this indicates that the isopropyl group of Cpd-2 likely binds more favourably to TDO2 than the n-propyl of Cpd-1. Potential reasons for this may include an improved shape fit of the branched isopropyl group, a reduced entropic penalty for the isopropyl group compared to the more flexible n-propyl group, and potential effects of both groups on the conformation of the free (unbound) inhibitor. Figure S6 online shows TDO2 residues adjacent to the isopropyl group of Cpd-2.

**Fig. 5:**
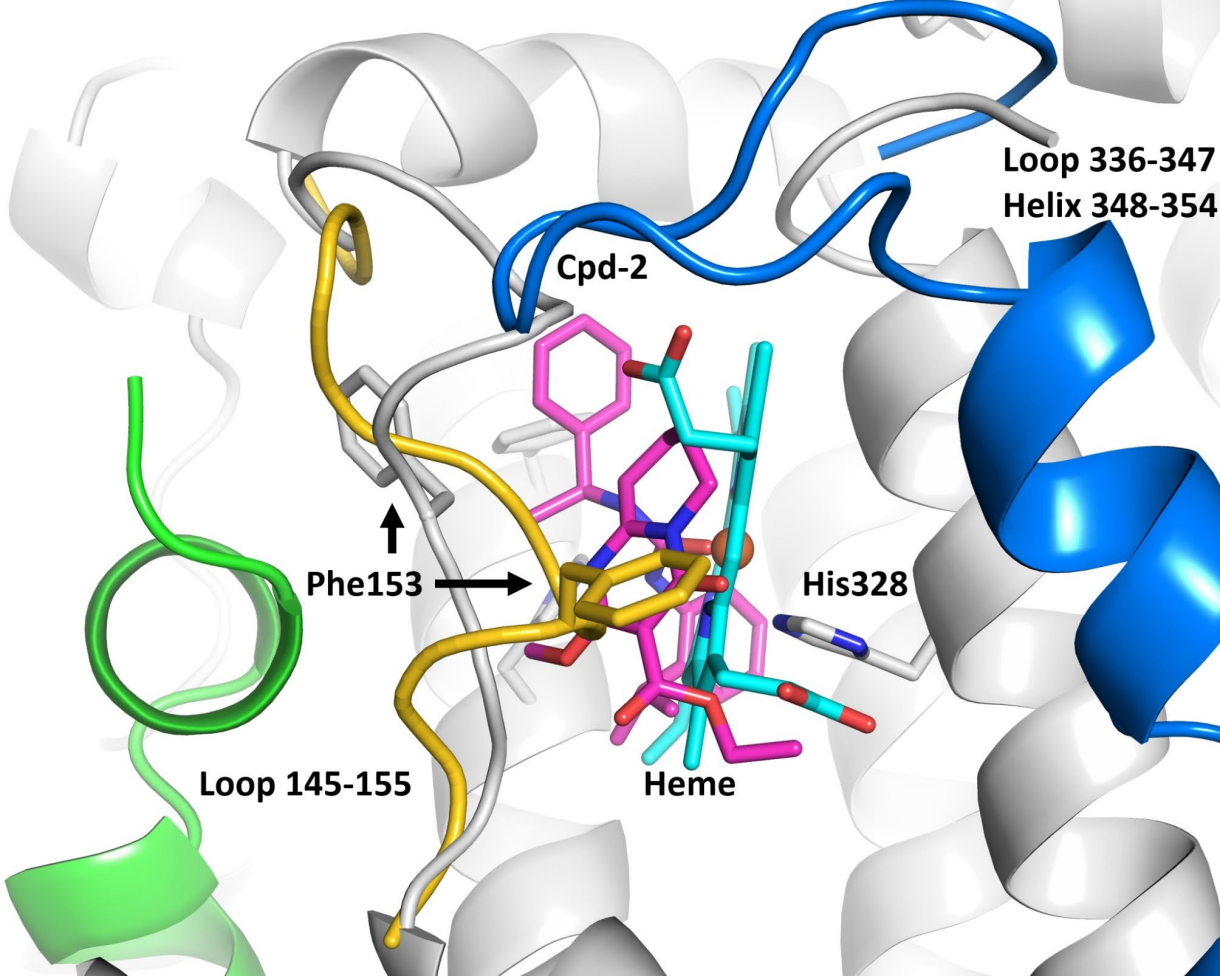
Superposition of the apo-TDO2 Cpd-2 complex and a holo-TDO2 Trp complex. The apo-TDO2 inhibitor complex is shown with protein molecules in green and grey cartoon, with Cpd-2 as sticks in magenta. The overlapping heme of holo-TDO2 (PDB ID 5TIA, Trp substrate omitted for clarity) is shown in cyan. Loop 145–155 of the superimposed holo-TDO2 structure shown in yellow. Residues 336–354 of holo-TDO2, which are disordered in both apo-TDO2 structures, are shown as cartoon in blue. In stick format, atoms are colored blue (N), red (O) and brown (Fe).

A superposition of the Cpd-2 apo-TDO2 complex with the structure of holo-TDO2 in complex with its substrate Trp (PDB ID 5TIA)^24^ is shown in Fig. 5. This highlights the extensive conformational change of the flexible loop 148–156 between apo-TDO2 and holo-TDO2 complexes (r.m.s.d. of 4.1 Å for the C_α_ atoms of residues 148–156), with C_α_ of Gly152 and Phe153 shifted by approximately 6 Å. Residues 336–354 of holo-TDO2, which are disordered in the apo-TDO2 structures, are shown in blue.

To allow a direct comparison of the structures of apo-TDO2 inhibitor complexes with uninhibited structures, all with AMT bound in the exosite, the structure of AMT bound to uninhibited apo-TDO2 was also solved (PDB 8QV7, space group C2). This structure is very similar to the published structure of apo-TDO2 without AMT (PDB entry ID 4PW8, space group P2_1_2_1_2_1_)^25^ apart from a small conformational shift of residues 148–152. The binding of AMT to the apo-TDO2 exosite (not shown) matches the published binding mode of tryptophan to the holo-TDO2 exosite^24^. AMT was also observed in the structure of Cpd-2 bound to apo-TDO2, with an identical binding mode.

## Discussion

We describe, for the first time to our knowledge, inhibitors binding to the apo form of TDO2 and inhibiting cellular TDO2 activity, together with the co-crystal structure of an inhibitor bound to apo-TDO2. While a former virtual screening to identify novel IDO1/TDO2 dual inhibitor scaffolds indicated that one of the hits might preferentially bind to the apo enzyme as revealed by molecular docking, no experimental evidence to support the finding was provided^26^. We believe that targeting the apo form of TDO2 offers a promising alternative approach for the development of therapeutic TDO2 inhibitors. Targeted inhibition of the apo form has been applied successfully to the structurally unrelated heme enzyme IDO1^23^, which together with TDO2 catalyzes the rate limiting first step of the kynurenine pathway. A superposition of the apo-IDO1 and apo-TDO2 inhibitor complexes is shown in Supplementary Fig. S3 online, showing the low structural similarity between the active sites and the unrelated binding mode and chemical structures of their inhibitors.

Rational holo-TDO2 inhibitor development is hampered by a poor correlation between biochemical and cellular assay potencies, with inhibitors reported thus far showing limited cellular potency^15^. As demonstrated by the high potency of Cpd-2, targeting the apo form of the enzyme offers the possibility to generate more potent TDO2 inhibitors both as tools to study the kynurenine pathway and as potential therapeutics. Instead of competing with the Trp substrate, apo-TDO2 inhibitors compete with the insertion of the heme cofactor itself. The mechanism, timing, and reversibility of heme insertion into TDO2 remain poorly understood and require further investigation^1,2^. Heme lability has been proposed as a posttranslational regulatory mechanism of both IDO1 and TDO2^1^. Free heme is present in the cells at very low concentrations (nM to single-digit µM concentrations)^27^ while intracellular Trp concentrations have been reported in the range of 0.1–0.6 mM^28,29^, offering a competitive advantage to apo- as compared to holo-binders. Another potential benefit of inhibitors targeting apo-TDO2 is their stabilization of the apo form, unlike holo-TDO2 inhibitors which likely shift the balance of cellular TDO2 towards the holo form. Following elimination of an apo-TDO2 inhibitor, we anticipate a longer recovery period for cellular TDO2 activity when heme (re)binding is required, as described for IDO1.

Our work has several limitations which need to be addressed with further experiments. The kinetics of heme association with (or active insertion into) TDO2 and its dissociation rate in living cells needs to be further explored, in a similar way to the recent studies on cytochrome P450 enzyme maturation^30^. The balance of apo- vs. holo-TDO2 in different cells and disease states requires further investigation. The development of optimized, potent apo-TDO2 inhibitors and investigation of their in vivo efficacy will allow us to further evaluate the apo form of TDO2 as a target for small molecule therapeutics.

## Methods

### Production of human apo-TDO2

To produce truncated TDO2 as the apo form devoid of heme, recombinant protein expression was carried out using an *E. coli hemB* (porphobilinogen synthase) mutant strain unable to synthesize heme (RP523, *E. coli* Genetic Stock Center, Yale CGSC#7199). *E. coli* RP523 carries an uncharacterized permeability mutation that renders the bacteria heme-permeable, allowing aerobic propagation in supplementing growth media containing hemin [30 µg/ml]^31,32^.

DNA encoding a truncated version of human TDO2 (P48775.1, amino acid (aa) 39–389) with a C-terminal hexa-histidine tag was codon optimized for expression in *E. coli* and synthesized by GeneArt (Thermo Fisher Scientific). The TDO2 construct was provided as an insert in the pMA-T vector backbone with NdeI and BamHI as flanking restriction sites for subcloning into the pDSNdeI (AMP^r^) protein expression vector^33^. The aa 39–389 truncation was designed based on the clearly ordered region of the holo-TDO2 structure (PDB entry 5TIA)^24^, with the aim of improving the expression yield and the likelihood of crystallization by removal of flexible termini.

The pDSNdeI derivative encoding the TDO2 construct was transformed into RP523 pRep4 (*lacI*,* Kan*^*r*^). RP523 pRep4 pDSNdeI::TDO2 aa39-389-His6 (NdeI, BamHI) was first grown aerobically (Luria-Bertani (LB) medium, ampicillin 100 µg/mL, kanamycin 25 µg/mL, 0.2% glucose, 2 µg/ml hemin, 37 ºC, 225 rpm, Kühner Labtherm incubator) to prepare an inoculum containing sufficient cells for anaerobic expression. Inocula prepared under aerobic conditions (2 µg/mL hemin) were harvested and resuspended in medium without hemin to minimize transfer of residual hemin to anaerobic expression cultures.

The medium for anaerobic protein expression (6 × 1 L LB, ampicillin 100 µg/mL, kanamycin 25 µg/mL, 0.2% glucose distributed to 4 screw cap flasks with magnetic stirrer) was equilibrated in an anaerobic glove box (Airlock, Coy Laboratory). The bacterial expression cultures were grown in the screw capped tightly closed flasks with moderate stirring. Protein expression was induced in the anaerobic glove box (100 µg/mL isopropyl ß-D-1-thiogalactopyranoside (IPTG)) at an optical density at 600 nm wavelength (OD_600_) of approximately 0.5. 50 µg/ml of Trp was added as additional supplement. Cultures were further grown overnight at 16 ºC with moderate stirring.

Expressing cells were harvested by centrifugation using a Fiberlite F12-6 × 500 Lex Rotor with a Sorvall RC6 + centrifuge (20 min, 6000 revolutions per minute (rpm) (6371 *g*), 4 ºC) and the bacterial cell pellets were stored at -70 ºC.

### Purification of human apo-TDO2 proteins

Bacterial cell pellets of expression cultures (~ 19.3 g) were thawed on ice and resuspended in lysis buffer (50 mM Tris-HCl pH 8.0, 400 mM NaCl, 1 mM TCEP, 5% glycerol, EDTA free completed protease inhibitor (Roche), 0.2 mg/ml lysozyme, 25 U/ml Benzonase nuclease, 10 mM MgCl_2_) for IMAC (immobilized metal (Ni) affinity chromatography). Cell breakage was carried out by high pressure homogenization (29008 pounds per square inch (psi), equal to 200 MPa) using a fluidizer (Microfluidics MP110P with DIXC H10Z chamber). Bacterial cell lysate was cleared by centrifugation (30 min at 16000 rpm (30392 *g*), Fiberlite F21-8 × 50y rotor) and applied to a 5 ml HisTrap Column (VWR, Cytiva) mounted on an Aekta Purifier 100 FPLC system.

Protein was eluted with a linear gradient of increasing imidazole (0-500 mM) concentration over 20 column volumes with buffer (50 mM Tris-HCl pH 8.0, 400 mM NaCl, 1 mM TCEP, 5% glycerol, 500 mM imidazole). Fractions (4 ml) were monitored for presence of TDO2 by SDS-PAGE (calculated molecular mass 42940.24 Da; theoretical pI 7.09, EXPASY protein parameter calculator https://web.expasy.org/protparam). Eluate fractions were analyzed by SDS-PAGE and immunoblot using an anti-histidine tag antibody (Monoclonal Mouse IgG1 histidine tag antibody; R&D Systems, cat. no. MAB050). Fractions containing TDO2 were combined and dialyzed (molecular weight cutoff 3.5 kDa, Spectrum^™^, 132725) overnight to remove imidazole (50 mM Tris-HCl pH 8.0, 50 mM NaCl, 1 mM TCEP, 5% glycerol).

The protein was further purified by anion exchange chromatography (HiTrap Q, VWR 17-1154-01) with a linear gradient of increasing NaCl salt concentration. Eluate fractions containing TDO2 were combined and concentrated to a protein concentration of 2 mg/ml (Amicon, molecular weight cutoff 10 kDa). Buffer exchange of the concentrated TDO2 sample was carried out by size exclusion chromatography using a HiLoad 16/60 Superdex 200 column equilibrated with running buffer (50 mM HEPES-NaOH, pH 8.0, 200 mM NaCl, 2 mM TCEP, 10% glycerol).

Protein concentration was determined spectrophotometrically using the absorbance at 280 nm and the calculated extinction coefficient. The same methods were used to express and purify a longer construct (His6-aa19-406, with aa406 corresponding to the wild type C-terminal residue), which was used for DSF and other experiments.

### Production of human holo-TDO2

Human holo-TDO2 was produced in a similar way to the previously described procedure^24^. Briefly, a construct encoding amino acids 19–406 of human TDO2 with an N-terminal six histidine tag linked by a PreScission protease cleavage site was expressed in *E. coli* BL21(DE3) cells. Following cell disruption and centrifugation, the supernatant was purified by nickel affinity chromatography (HisTrap HP), followed by buffer exchange and anion exchange purification (HiTrap Q). The pooled eluate fractions were concentrated and purified by size exclusion chromatography (HiLoad 16/60 Superdex 200). All chromatography steps were carried out using an Äkta Purifier. The purified protein in a final buffer of 50 mM sodium phosphate pH 8.0, 300 mM NaCl, 5% glycerol was stored at -80 °C for use in enzymatic assays.

### Differential scanning fluorimetry

Protein thermal stability and small molecule ligand binding of TDO2 in vitro was studied using differential scanning fluorimetry (DSF, also known as thermal shift assay)^34^. Assay ingredients and ligands were combined to obtain a mixture composed of 10 µM TDO2 and 10x SYPRO Orange, in assay buffer (50 mM HEPES-NaOH pH 8.0, 200 mM NaCl, 2 mM TCEP, 10% glycerol) and 2% final DMSO concentration. Small molecule chemical ligands were taken from DMSO stock solution (100 mM, 10 mM) and diluted in DMSO transferred to wells of PCR Plates (Micro Amp Fast 96-well Reaction Plates, Applied Biosystems) to ensure matching final DMSO concentrations.

DSF was carried out with a StepOnePlus™ real time PCR system. Thermal denaturation of TDO2 was monitored with a ramp rate of 1 K/min. Protein melting curves were evaluated using the Thermal Shift Software v 1.3. (Thermo Fisher Scientific) to derive differences in midpoint melting-temperatures (derivative model). The thermal shift (DTm) is documented as the difference between the reference midpoint melting temperature of proteins (containing 500 µM AMT) in the presence of the ligand vehicle (DMSO) and in the presence of ligand.

### Holo-TDO2 UV-VIS absorbance

A UV − visible spectra study was performed to evaluate the binding of the compounds. The holo-TDO2 absorbance spectra were measured using a BioTek Synergy multimode reader (Agilent) for wavelengths from 250 to 600 nm in 1 nm increments. 5 µM holo-TDO2 was incubated at 37 °C with 20 µM compounds or DMSO in phosphate buffer supplemented with 0.01% Tween-20 in a 96 well UV compatible plate (Corning #3679) and absorbance was measured before (t = 0 h) and after 2–6 h of incubation.

### Crystallization and structure determination

All crystals were grown using the sitting drop vapour diffusion method at 293 K. The addition of AMT to apo-TDO2 was essential to obtain high quality crystals.

#### Apo-TDO2 with AMT

Truncated apo-TDO2 at a concentration of 20.5 mg/ml (480 µM) containing 2 mM AMT was crystallized using 50 mM HEPES-NaOH pH 8.0, 200 mM NaCl, 2 mM TCEP and 10% glycerol as a reservoir solution.

#### Apo-TDO2 with AMT and Cpd-2

Truncated apo-TDO2 at a concentration of 5.3 mg/ml (124 µM) containing 2 mM AMT, was crystallized after incubation with 1 mM inhibitor from a 10 mM DMSO stock for two hours. The reservoir solution contained 10% (w/v) PEG 4000, 20% (v/v) glycerol, 0.03 M each of sodium nitrate, disodium hydrogen phosphate, ammonium sulfate, 0.1 M MES/imidazole pH 6.5 (condition C3 of the Morpheus I Screen)^35^.

### X-ray data collection and crystal structure determination

Crystals were harvested in loops and cryocooled directly in liquid nitrogen. Data collection was carried out at beamline X06DA/PXIII of the Swiss Light Source, Villigen, Switzerland. X-ray diffraction data were processed using autoPROC^36^. The structures were solved using Dimple and Phaser^37^ for automated molecular replacement, Coot^38^ for rebuilding and superpositioning, and BUSTER^39^ for structure refinement. Ligand constraint files were created using Grade2 ^40^. Figures were created using PyMOL^41^. BUSTER-report was used for analysis of ligand geometry and fit to the electron density, including the comparison of both possible stereoisomers of Cpd-2. The data processing and refinement statistics are shown in Supplementary Table S1 online.

The structures were deposited in the Protein Data Bank (PDB) with entry IDs 8QV7 (AMT) and 9EZJ (AMT and Cpd-2). The raw data (diffraction images) used for both structures were deposited at proteindiffraction.org and are accessible via the PDB entry.

### Assays

#### TDO2 cellular activity assay

SW48 cells (source: American Type Culture Collection (ATCC), CCL-231) were used to measure the TDO2 inhibitory activity of compounds and were routinely maintained in DMEM high glucose/GlutaMAX^TM^/pyruvate (Gibco, 10569010) 90% (v/v), FCS (Gibco, 10500, heat inactivated)10% (v/v), Penicillin/streptomycin (Gibco, 15140) 1% (v/v). SW48 cells were seeded in 384 well plates at a density of 8000 cells in 45 µl per well. Plates were incubated at 37 °C / 5% CO_2_ for 24 h.

On the next day, 10 µl compound in serial dilutions (tested concentration range 0.5 nM to 10 µM) and 200 µM Trp were added. After 24 h of incubation at 37 °C / 5% CO_2_, 3 µl of the supernatant per well was transferred into 25 µl water per well in a 384 deep well plate.

The 384 deep well plate containing 3 µl supernatant and 25 µl H_2_O per well were further processed for LCMS. After the addition of 100 µl Trp-(indole-d5) (Sigma 615862) at 200 nM in methanol, the 384 deep well plates were centrifuged for 10 min at 3220 *g* at 4 °C, 75 µl H_2_O was added per well and plates centrifuged again for 10 min at 3220 *g* at 4 °C. NFK and kynurenine were quantified by LCMS, normalized to the internal standard Trp-(indole-d5) and the sum was calculated. NFK is converted directly to kynurenine (KYN) by NFK formamidase.

Samples with 0.2% DMSO (0% effect) and the reference holo-TDO2 inhibitor 680C91^42^ (100% effect) were used as control samples to set the parameters for the non-linear regression necessary for the determination of the IC_50_ for each compound and the determination of the Z’ factor for quality control. For each compound concentration the percentage of activity compared to 0% and 100% effect was calculated as average ± standard deviation (each concentration measured in duplicate). IC_50_ values and curves were generated with XLfit software (IDBS) using Dose-Response One Site model 203 (4 Parameter Logistic Model. Parameter A locked to 0 and B to 100). A serial dilution of the TDO2 inhibitor was included on each plate of the experiment and its IC50 was calculated. In the reported experiments, the deviation factor of the IC_50_ value of the reference TDO2 inhibitor was < 3 versus the actual arithmetic mean and Z′ was > 0.5.

#### IDO1 cellular activity assay

SKOV3 cells (source: National Cancer Institute (NCI), No. 0503405) which upregulate IDO1 after stimulation with IFNγ were used to measure compounds for IDO1 inhibitory activity^43^. SKOV3 cells were routinely maintained in RPMI 90% (v/v), FCS 10% (v/v), Penicilin/streptomycin 1% (v/v). SKOV3 cells were seeded in 384 well plates at a density of 8000 cells in 45 µl per well or 4000 cells in 45 µl per well, respectively. Plates were incubated at 37 °C / 5% CO2 for 24 h. On the next day, 10 µl compound in serial dilutions (tested concentration range 10 µM-0.5 nM) and 200 µM L-tryptophan were added as well as IFNγ at a final concentration of 50 ng/ml. After 24 h of incubation at 37 °C / 5% CO2, 3 µl of the supernatant per well was transferred to 25 µl H_2_O per well in a 384 deep well plate and 25 µl of the supernatant per well was transferred to waste. The SKOV3 cell plates with 25 µl supernatant per well remaining were used to measure viability (see below). The 384-deep well plate containing 3 µl supernatant and 25 µl H_2_O per well were further processed for LCMS: After the addition of 100 µl L-tryptophan-(indole-d5) (Sigma, #615862) at 200 nM in methanol, the 384-deep well plates were centrifuged for 10 min at 3220 x g at 4 °C, 75 µl H_2_O was added per well and plates centrifuged again for 10 min at 3220 x g at 4 °C. NFK and kynurenine (KYN) were quantified by LCMS, normalized to the internal standard L-tryptophan-(indole-d5) and the sum was calculated. Samples with 0.2% DMSO (0% effect) and IDO1 inhibitor (INCB14943, CAS 914471-09-3) (100% effect)^44^ were used as control samples to set the parameters for the non-linear regression necessary for the determination of the IC50 for each compound. For each compound concentration the percentage of activity compared to 0% and 100% effect was calculated as average ± STDEV (each concentration measured in duplicate). IC50 values and curves were generated with XLfit software (IDBS) using Dose-Response One Site model 203 (4 Parameter Logistic Model. Parameter A locked to 0 and B to 100).

#### Cell viability assay

As inhibition of NFK and KYN production can simply be an effect of cytotoxicity, a viability assay (CellTiter-Glo 2.0 Luminescent Cell Viability Assay, Promega Catalog # G9243) was performed in parallel. CellTiter-Glo reagent was added (25 µl/well) to SW48 cell plates, incubated for 15 min at room temperature in the dark and luminescence was measured with the EnVision Multilabel Reader from Perkin Elmer according to the manufacturer’s instructions. The luminescent signal is proportional to the amount of ATP present. The amount of ATP is directly proportional to the number of viable cells present. Samples with 0.2% DMSO (0% effect) and a toxic reference compound (100% effect) were used as control samples to set the parameters for the non-linear regression. For each compound concentration the percentage of activity compared to 0% and 100% effect was calculated as average ± STDEV (each concentration measured in duplicate). Cytotoxicity IC_50_ values and curves were generated with XLfit software (IDBS) using Dose-Response One Site model 203 (4 Parameter Logistic Model with parameter A locked to 0 and B to 100).

#### Holo-TDO2 enzymatic assay

For the enzymatic assay, recombinant human TDO2 comprising amino acids 19–407 with a N-terminal hexahistidine tag, expressed in *Escherichia coli* and purified to homogeneity, was incubated in assay buffer consisting of 75 mM phosphate buffer at pH 7.0 supplemented with 100 µM ascorbic acid, 50 U/ml catalase, 0.01% bovine serum albumin (BSA), and 0.01% Tween 20 (protocol modified from^45^).

### Synthesis of Cpd-1 and Cpd-2

The synthesis and spectroscopic analysis of Cpd-1 and Cpd-2 are described in the Supplementary Information online.

## Electronic supplementary material

Below is the link to the electronic supplementary material.

Supplementary Material 1

## Data Availability

The datasets generated and analysed during the current study are available in the PDB repository, with accession codes 8QV7 (apo-TDO2 with AMT) and 9EZJ (apo-TDO2 with AMT and Cpd-2). Raw diffraction data are available at https://proteindiffraction.org using the PDB accession codes.

## Abbreviations

AMT: alpha-methyl-L-tryptophan
BSA: bovine serum albumin
DSF: differential scanning fluorimetry
GAPDH: glyceraldehyde-3-phosphate dehydrogenase
IDO1: indoleamine-2,3-dioxygenase
KYN: kynurenine
LB: Luria-Bertani
NFK: N-formyl-kynurenine
NO: nitric oxide
psi: pounds per square inch
rpm: revolutions per minute
TDO2: tryptophan-2,3-dioxygenase
Trp: L-tryptophan
