# Supplementary material for: Discovery and binding mode of small molecule inhibitors of the apo form of human TDO2
Source: Sci Rep. 2024 Nov 14;14:27937. doi: 10.1038/s41598-024-78981-4 (PMC11561238; doi:10.1038/s41598-024-78981-4)
Supplement: Supplementary file 1 — Supplementary Material 1 [file 41598_2024_78981_MOESM1_ESM.docx]

Title: Discovery and binding mode of small molecule inhibitors of the apo form of human TDO2

Carina Lotz-Jenne^¶*^, Roland Lange^¶^, Sylvaine Cren, Geoffroy Bourquin, Laksmei Goglia, Thierry Kimmerlin, Micha Wicki, Manon Müller, Nadia Artico, Sabine Ackerknecht, Philippe Pfaff, Christoph Joesch and Aengus Mac Sweeney^*^

# Supplementary Information

## Protein Expression

aa39-389

MGLIYGNYLH LEKVLNAQEL QSETKGNKIH DEHLFIITHQ AYELWFKQIL WELDSVREIF

QNGHVRDERN MLKVVSRMHR VSVILKLLVQ QFSILETMTA LDFNDFREYL SPASGFQSLQ

FRLLENKIGV LQNMRVPYNR RHYRDNFKGE ENELLLKSEQ EKTLLELVEA WLERTPGLEP

HGFNFWGKLE KNITRGLEEE FIRIQAKEES EEKEEQVAEF QKQKEVLLSL FDEKRHEHLL

SKGERRLSYR ALQGALMIYF YREEPRFQVP FQLLTSLMDI DSLMTKWRYN HVCMVHRMLG

SKAGTGGSSG YHYLRSTVSD RYKVFVDLFN LSTYLIPRHW IPKMNPTIHK FL**EHHHHHH**

aa19-406

**MRGSHHHHHH** GIDHMPVEGS EEDKSQTGVN RASKGGLIYG NYLHLEKVLN AQELQSETKG

NKIHDEHLFI ITHQAYELWF KQILWELDSV REIFQNGHVR DERNMLKVVS RMHRVSVILK

LLVQQFSILE TMTALDFNDF REYLSPASGF QSLQFRLLEN KIGVLQNMRV PYNRRHYRDN

FKGEENELLL KSEQEKTLLE LVEAWLERTP GLEPHGFNFW GKLEKNITRG LEEEFIRIQA

KEESEEKEEQ VAEFQKQKEV LLSLFDEKRH EHLLSKGERR LSYRALQGAL MIYFYREEPR

FQVPFQLLTS LMDIDSLMTK WRYNHVCMVH RMLGSKAGTG GSSGYHYLRS TVSDRYKVFV

DLFNLSTYLI PRHWIPKMNP TIHKFLYTAE YCDSSYFSSD ESD

**Amino acid sequence of short (aa39-389) and long (aa19-406) TDO**. Colour code: black, TDO2 sequence; blue, additional amino acids introduced as a linker or during subcloning; red, 6 histidine tag.

## Synthesis of Cpd-1 and Cpd-2

**General.** All reagents and solvents are used as supplied. Air- and moisture-sensitive reactions are carried out under inert atmosphere. The reaction time and temperature (RT: room temperature) are indicative. Progress of the reactions is followed either by thin-layer chromatography (TLC) analysis (Merck, silica gel 60 F_254_ TLC plates) or by liquid chromatography-mass spectrometry (LC-MS). LC-MS conditions (acidic, Zorbax): Thermo Finnigan MSQ Plus, with Waters iClass BSM Binary Pump and DAD Waters iClass PDA; column: Zorbax RRHD SB-Aq, 2.1 x 50 mm, 1.8 μm (Agilent); gradient: 5-95% acetonitrile in water containing 0.04% of trifluoroacetic acid (TFA) over 1.20 min, then 95% acetonitrile in water containing 0.04% of TFA for 0.70 min; flow: 0.8 mL/min; temperature: 40 °C; retention time (*t*_R_) is given in min.

Compounds are purified either by flash column chromatography (FC) on silica gel 60 using solvents such as Hept (heptane), EtOAc (ethyl acetate), CH_2_Cl_2_ or MeOH (methanol), or by reverse phase preparative high-performance liquid chromatography (prep-HPLC) using Waters XBridge C18 column (10 μm, 75 x 30 mm) with eluents: A, water + 0.5% acidic or basic additive; B, acetonitrile; detection: UV/vis and/or MS and/or ELSD. Prep-HPLC (acidic conditions): additive in A is HCO_2_H. Prep-HPLC (basic conditions): additive in A is NH_4_OH.

Title compounds are analyzed by LC-MS (see conditions above) and ^1^H NMR (nuclear magnetic resonance) spectroscopy. In addition, final compounds Cpd-1 and Cpd-2 are analyzed by high resolution LC-MS (LC-HRMS). LC-HRMS conditions: UPLC SYNAPTG2 Q-tof, with Waters Acquity Binary Analytical Pump and DAD Acquity UPLC PDA Detector; column: Acquity UPLC CSH C18 1.7 μm 2.1 x 50 mm (Waters); gradient: 2-98% acetonitrile containing 0.045% of formic acid in water containing 0.05% of formic acid over 2.0 min; flow: 1.0 mL/min; detection: UV 214 nm and MS; temperature: 60 °C; *t*_R_ is given in min; [M+H^+^]/z calculated (calcd.) and found. ^1^H and ^13^C NMR (nuclear magnetic resonance) spectra are recorded on a Bruker Avance II 400 MHz or Bruker Ascend 500 MHz spectrometer in the indicated deuterated solvent. Chemical shifts are reported in parts per million (ppm) relative to solvent peaks as the internal reference. Multiplicities are given as s (singlet), d (doublet), t (triplet), q (quartet), p (pentet), hept (heptet) or m (multiplet). Coupling constants are given in Hz.

**Ethyl 2-methoxy-4-oxo-9-(1-(1-phenylethyl)-3-(2-propylphenyl)ureido)-6,7,8,9-tetrahydro-4*H*-pyrido[1,2-*a*]pyrimidine-3-carboxylate (Cpd-1)**

**Step 1: ethyl 2-hydroxy-4-oxo-4*H*-pyrido[1,2-*a*]pyrimidine-3-carboxylate** (procedure adapted from patent priority application FR2198697)

A mixture of 2-aminopyridine (2401 mg, 25 mmol, 1 eq) and triethyl methanetricarboxylate (10.6 mL, 50 mmol, 2 eq) in bromobenzene (200 mL) is stirred at 156 °C for 18 h. The reaction mixture is cooled to RT and bromobenzene removed under reduced pressure. The residue is suspended in Et_2_O, filtered and the solid dried under reduced pressure to give the title compound as a mixture of compounds (3.58 g) which is used in the next step without further purification. LC-MS (acidic, Zorbax) *t*_R_ = 0.42 min, [M+H]^+^ = 235.10. ^1^H NMR (500 MHz, DMSO) δ 12.41 (s, 1H), 8.93 (dd, *J* = 7.4, 1.6 Hz, 1H), 8.19 (ddd, *J* = 8.7, 6.9, 1.6 Hz, 1H), 7.42 – 7.36 (m, 2H), 4.15 (q, *J* = 7.2 Hz, 2H), 1.24 (t, *J* = 7.1 Hz, 3H).

**Step 2: ethyl 2-methoxy-4-oxo-4*H*-pyrido[1,2-*a*]pyrimidine-3-carboxylate**

Ethyl 2-hydroxy-4-oxo-4*H*-pyrido[1,2-*a*]pyrimidine-3-carboxylate (2562 mg, 10.9 mmol, 1 eq) is suspended in toluene (75 mL). (Trimethylsilyl)diazomethane solution 2.0 M in hexanes (7.2 mL, 14.4 mmol, 1.3 eq) is added dropwise at RT and the reaction mixture stirred at RT for 1 h. MeOH (25 mL) is carefully added (gas evolution) and the mixture stirred at RT for 1 h before concentrating under reduced pressure. The crude residue is purified by prep-HPLC (acidic conditions) to give the title compound as a white solid (1.36 g). LC-MS (acidic, Zorbax) *t*_R_ = 0.69 min, [M+H]^+^ = 249.34. ^1^H NMR (500 MHz, DMSO) δ 8.97 (ddd, *J* = 7.0, 1.7, 0.9 Hz, 1H), 8.10 (ddd, *J* = 8.5, 6.8, 1.6 Hz, 1H), 7.64 (dt, *J* = 8.9, 1.2 Hz, 1H), 7.43 (td, *J* = 6.9, 1.4 Hz, 1H), 4.24 (q, *J* = 7.1 Hz, 2H), 3.98 (s, 3H), 1.27 (t, *J* = 7.1 Hz, 3H). ^13^C NMR (126 MHz, DMSO) δ 165.08, 164.54, 156.00, 150.57, 140.91, 128.80, 125.07, 117.09, 92.78, 60.91, 54.75, 14.57.

**Step 3: ethyl 2-methoxy-4-oxo-6,7,8,9-tetrahydro-4*H*-pyrido[1,2-*a*]pyrimidine-3-carboxylate**

Ethyl 2-methoxy-4-oxo-4*H*-pyrido[1,2-*a*]pyrimidine-3-carboxylate (1360 mg, 5.48 mmol, 1 eq) is dissolved in DMF (40 mL), the mixture inerted with N_2_ and palladium on activated carbon, moistened with water, 10% Pd basis (583 mg, 0.548 mmol, 0.1 eq) added. The reaction mixture is stirred at 50 °C under atmospheric H_2_ for 3 h. The mixture is cooled to RT, filtered and the filtrate concentrated under reduced pressure. The crude residue is purified by FC (CH_2_Cl_2_/MeOH) to give the title compound as a pale yellow solid (1.38 g). LC-MS (acidic, Zorbax) *t*_R_ = 0.67 min, [M+H]^+^ = 253.19. ^1^H NMR (400 MHz, DMSO) δ 4.17 (q, *J* = 7.1 Hz, 2H), 3.86 (s, 3H), 3.76 (t, *J* = 6.1 Hz, 2H), 2.85 (t, *J* = 6.6 Hz, 2H), 1.87 (p, *J* = 5.5 Hz, 2H), 1.84-1.73 (m, 2H), 1.22 (t, *J* = 7.1 Hz, 3H).

**Step 4: ethyl 9-bromo-2-methoxy-4-oxo-6,7,8,9-tetrahydro-4*H*-pyrido[1,2-*a*]pyrimidine-3-carboxylate**

A mixture of ethyl 2-methoxy-4-oxo-6,7,8,9-tetrahydro-4*H*-pyrido[1,2-*a*]pyrimidine-3-carboxylate (1378 mg, 5.46 mmol, 1 eq), *N*-bromosuccinimide (982 mg, 5.46 mmol, 1 eq) and 2,2'-azobis(2-methylpropionitrile) (91.5 mg, 0.546 mmol, 0.1 eq) in CHCl_3_ (15 mL) is stirred at RT for 24 h. The solvent is removed under reduced pressure and the residue purified by FC (Hept/EtOAc). The fractions are concentrated under reduced pressure, the residue triturated with Et_2_O, filtered and the filtrate concentrated under reduced pressure to give the title compound as a yellow oil (1.36 g). LC-MS (acidic, Zorbax) *t*_R_ = 0.79 min, [M+H]^+^ = 331.18. ^1^H NMR (500 MHz, CDCl3) δ 5.14 (ddd, *J* = 4.1, 2.5, 1.3 Hz, 1H), 4.38 (q, *J* = 7.2 Hz, 2H), 4.36-4.29 (m, 1H), 3.99 (s, 3H), 3.77 (ddd, *J* = 15.0, 11.0, 6.0 Hz, 1H), 2.50-2.35 (m, 2H), 2.24 (ddt, *J* = 15.1, 12.1, 4.0 Hz, 1H), 2.18-2.07 (m, 1H), 1.38 (t, *J* = 7.2 Hz, 3H). ^13^C NMR (126 MHz, CDCl_3_) δ 165.86, 164.05, 160.47, 158.17, 98.79, 77.29, 77.03, 76.78, 61.38, 55.10, 44.73, 42.43, 28.60, 17.57, 14.23.

**Step 5: ethyl-2-methoxy-4-oxo-9-((1-phenylethyl)amino)-6,7,8,9-tetrahydro-4*H*-pyrido[1,2-*a*]pyrimidine-3-carboxylate**

Ethyl 9-bromo-2-methoxy-4-oxo-6,7,8,9-tetrahydro-4*H*-pyrido[1,2-*a*]pyrimidine-3-carboxylate (610 mg, 1.84 mmol, 1 eq) is dissolved in DMF (18 mL). Et_3_N (0.28 mL, 2.03 mmol, 1.1 eq) is added at RT, followed by alpha-methylbenzylamine (0.50 mL, 3.87 mmol, 2.1 eq). The reaction mixture is stirred at RT for 1h30 and concentrated under reduced pressure. The crude residue is purified by FC (CH_2_Cl_2_/MeOH) to give the title compound as an orange oil (536 mg, mixture of 4 stereoisomers). LC-MS (acidic, Zorbax) *t*_R_ = 0.62 and 0.63 min, [M+H]^+^ = 372.46. ^1^H NMR (400 MHz, DMSO) δ 7.41 (ddd, *J* = 8.1, 4.9, 1.4 Hz, 2H), 7.37-7.28 (m, 2H), 7.28-7.17 (m, 1H), 4.30 (q, *J* = 6.5 Hz, 1H), 4.23-4.12 (m, 2H), 3.96 and 3.92 (s, 3H), 3.86-3.65 (m, 1H), 3.58-3.45 (m, 1H), 3.33-3.25 (m, 1H), 2.02-1.59 (m, 4H), 1.34-1.16 (m, 6H), NH not visible.

**Step 6: ethyl 2-methoxy-4-oxo-9-(1-(1-phenylethyl)-3-(2-propylphenyl)ureido)-6,7,8,9-tetrahydro-4*H*-pyrido[1,2-*a*]pyrimidine-3-carboxylate (Cpd-1)**

Ethyl-2-methoxy-4-oxo-9-((1-phenylethyl)amino)-6,7,8,9-tetrahydro-4*H*-pyrido[1,2-*a*]pyrimidine-3-carboxylate (100 mg, 0.27 mmol, 1 eq) is dissolved in DMF (2 mL). 1-Isocyanato-2-propylbenzene (91 mg, 0.54 mmol, 2 eq) is added at RT and the reaction mixture stirred at 60 °C for 5 h. The mixture is cooled to RT then filtered and concentrated under reduced pressure. The crude residue is purified by prep-HPLC (basic conditions) to give the title compound as a white solid (44 mg, mixture of 4 stereoisomers). LC-MS (acidic, Zorbax) *t*_R_ = 1.06 min, [M+H]^+^ = 533.32. LC-HRMS *t*_R_ = 1.253 min, [M+H^+^]/z calcd. for C_30_H_36_N_4_O_5_, 533.2758; found 533.2768. ^1^H NMR (500 MHz, DMSO) δ 8.33 and 7.99 (s, 1H), 7.77-7.66 (m, 1H), 7.57-7.50 (m, 1H), 7.50-7.26 (m, 3H), 7.24-7.00 (m, 4H), 5.56 and 5.47 (d, *J* = 5.8 and 6.9 Hz, 1H), 4.37-4.28 (m, 1H), 4.24-4.13 (m, 2H), 4.13-4.03 (m, 2H), 3.92 and 3.49 (s, 3H), 3.27-3.05 (m, 1H), 2.30-2.09 (m, 1H), 2.07-1.92 (m, 1H), 1.89-1.71 (m, 2H), 1.82 and 1.74 (d, *J* = 6.7 and 7.0 Hz, 3H), 1.60-1.39 (m, 3H), 1.21 and 1.17 (t, *J* = 7.1 Hz, 3H), 0.88 and 0.83 (t, *J* = 7.3 Hz, 3H).

**Ethyl-9-(3-(2-isopropylphenyl)-1-((*S*)-1-phenylethyl)ureido)-2-methoxy-4-oxo-6,7,8,9-tetrahydro-4*H*-pyrido[1,2-*a*]pyrimidine-3-carboxylate (Cpd-2)**

**Step 1: ethyl-2-methoxy-4-oxo-9-(((*S*)-1-phenylethyl)amino)-6,7,8,9-tetrahydro-4*H*-pyrido[1,2-*a*]pyrimidine-3-carboxylate**

Ethyl 9-bromo-2-methoxy-4-oxo-6,7,8,9-tetrahydro-4*H*-pyrido[1,2-*a*]pyrimidine-3-carboxylate (as prepared for the synthesis of Cpd-1, step 4) (500 mg, 1.41 mmol, 1 eq) is dissolved in DMF (7 mL). Et_3_N (0.22 mL, 1.55 mmol, 1.1 eq) is added at RT, followed by (*S*)-alpha-methylbenzylamine (0.37 mL, 2.82 mmol, 2.0 eq). The reaction mixture is stirred at RT for 2h30 and concentrated under reduced pressure. The crude residue is purified by FC (CH_2_Cl_2_/MeOH) to give the title compound as a yellow oil (606 mg, mixture of 2 stereoisomers). LC-MS (acidic, Zorbax) *t*_R_ = 0.63 and 0.64 min, [M+H]^+^ = 372.43. ^1^H NMR (400 MHz, DMSO) δ 7.46-7.37 (m, 2H), 7.37-7.28 (m, 2H), 7.27-7.17 (m, 1H), 4.37-4.25 (m, 1H), 4.25-4.10 (m, 2H), 3.96 and 3.92 (s, 3H), 3.86-3.65 (m, 1H), 3.58-3.45 (m, 1H), 3.30 (d, *J* = 7.0 Hz, 1H), 2.03-1.58 (m, 4H), 1.35-1.17 (m, 6H), NH not visible.

**Step 2: ethyl-9-(3-(2-isopropylphenyl)-1-((*S*)-1-phenylethyl)ureido)-2-methoxy-4-oxo-6,7,8,9-tetrahydro-4*H*-pyrido[1,2-*a*]pyrimidine-3-carboxylate (Cpd-2)**

Ethyl-2-methoxy-4-oxo-9-(((*S*)-1-phenylethyl)amino)-6,7,8,9-tetrahydro-4*H*-pyrido[1,2-*a*]pyrimidine-3-carboxylate (41 mg, 0.10 mmol, 1 eq) is dissolved in DMF (1 mL). 1-Isocyanato-2-(propan-2-yl)benzene (34 mg, 0.20 mmol, 2 eq) is added at RT and the reaction mixture stirred at this temperature for 48 h. The mixture is filtered and concentrated under reduced pressure. The crude residue is purified by prep-HPLC (basic conditions) to give the title compound as a white solid (19 mg, mixture of 2 stereoisomers). LC-MS (acidic, Zorbax) *t*_R_ = 1.06 min, [M+H]^+^ = 533.35. LC-HRMS *t*_R_ = 1.235 min, [M+H^+^]/z calcd. for C_30_H_36_N_4_O_5_, 533.2758; found 533.2765. ^1^H NMR (500 MHz, DMSO) *δ*: 8.41 and 8.09 (broad s, 1H), 7.72 (broad d, *J* = 7.0 Hz) and 7.62 (dd, *J* = 8.0, 1.4 Hz, 1H), 7.55 (broad d, *J* = 7.0 Hz, 1H), 7.46 (dd, *J* = 8.3, 6.9 Hz, 1H), 7.44-7.21 (m, 3H), 7.2-6.99 (m, 3H), 5.56 and 5.48 (q, *J* = 6.1 and 6.8 Hz, 1H), 4.35-4.30 and 4.15-4.08 (m, 1H), 4.19-4.06 (m, 4H), 3.94 and 3.46 (s, 3H), 3.25-3.06 (m, 1H), 3.02 and 2.84 (hept, *J* = 6.9 and 6.7 Hz, 1H), 2.28-2.06 (m, 1H), 2.06-1.96 and 1.53-1.38 (m, 1H), 1.83 and 1.74 (d, *J* = 6.9 and 7.0 Hz, 3H), 1.82-1.75 (m, 1H), 1.20 and 1.17 (t, *J* = 7.1 Hz, 3H), 1.12 and 1.08 (d, *J* = 6.9 Hz, 3H), 1.03 and 0.94 (d, *J* = 6.9 Hz, 3H). ^13^C NMR (126 MHz, DMSO) δ 165.22, 164.98, 164.50, 164.37, 160.16, 160.14, 155.31, 155.15, 145.71, 145.22, 141.53, 141.40, 140.15, 136.58, 136.51, 136.22, 129.00, 128.80, 128.75, 128.63, 128.36, 128.24, 128.21, 127.97, 126.64, 126.45, 126.17, 126.03, 125.99, 125.92, 125.88, 125.70, 124.52, 124.39, 96.32, 96.21, 60.67, 60.61, 55.00, 54.85, 54.43, 54.31, 54.09, 53.99, 42.70, 42.65, 27.91, 27.84, 27.66, 27.32, 24.06, 23.88, 23.16, 22.93, 20.49, 20.37, 19.55, 17.88, 14.50, 14.46.

## X-ray data processing and refinement statistics

|  |  | **AMT** | **Cpd-2 / AMT** |
| --- | --- | --- | --- |
| **Data collection** | | | |
| Source |  | SLS X06DA | SLS X06DA |
| Space Group |  | C2 | P2_1_2_1_2 |
| Wavelength (Å) |  | 1.00 | 1.00 |
| Cell dimensions | a, b, c (Å)  α, β, γ (°) | 181.3, 90.8, 132.8  90.0, 120.8, 90.0 | 133.8, 156.2, 90.7  90.0, 90.0, 90.0 |
| Observed reflections |  | 130624 | 394839 |
| Unique reflections |  | 38179 | 57974 |
| Resolution  (highest shell) (Å) |  | 77.91-2.93  (2.98-2.93) | 45.33-2.61  (2.66-2.61) |
| Rpim (%) |  | 16.3 (90.9) | 15.2 (134.8) |
| Rmerge (%) |  | 25.7 (147.0) | 36.8 (330.0) |
| Mean I/σ(I) |  | 3.2 (0.8) | 4.7 (0.7) |
| Completeness (%) |  | 95.3 (100.0) | 100.0 (98.5) |
| Redundancy |  | 3.4 (3.6) | 6.8 (6.9) |
| CC_0.5_ |  | (0.35) | (0.32) |
| **Refinement** | | | |
| Resolution  (highest shell) (Å) |  | 41.4-2.93  (2.95-2.93) | 45.33-2.61  (2.87-2.61) |
| R_work_ (highest shell) (%) |  | 24.4 (33.6) | 30.0 (37.4) |
| R_free_ (highest shell) (%) |  | 26.9 (41.4) | 33.0 (48.2) |
| B-factors (Å^2^) (number of non-H atoms) | Protein | 54.0 (9930) | 38.7 (10055) |
|  | Ligands | 49.5 (64) AMT  46.0 (76) All ligands | 34.8 (156) Cpd-2  31.3 (64) AMT  33.4 (220) All ligands |
|  | Water | 24.8 (12) | 8.9 (8) |
| Rms deviation | Bond lengths (Å) | 0.011 | 0.007 |
|  | Bond angles (°) | 1.40 | 0.81 |
| Ramachandran plot (%) | Favoured | 97.5 | 96.8 |
|  | Allowed | 2.5 | 2.5 |
|  | Disallowed | 0.0 | 0.7 |
| PBD entry ID |  | 8QV7 | 9EZJ |

**Supplementary Table S1.** Data collection and refinement statistics for apo-TDO2, and for the Cpd-2 / apo-TDO2 complex. Both structures contain AMT bound at the exosite of TDO2. Ramachandran outliers in the Cpd-2 complex structure 9EZJ are Gly63 (chains A-D), Ser148 and Gly152 (chains A and B) and Gly337 (chain A).

|  | **Tm Boltzmann** | **Δ Tm Boltzmann** | **Boltzmann fit** | **Tm Derivative** | **Δ Tm Derivative** |
| --- | --- | --- | --- | --- | --- |
| **Apo** | 48.68 |  |  | 48.51 |  |
| **Apo** | 48.9 |  |  | 49.23 |  |
| **Apo** | ***50.02*** |  |  |  |  |
| **Apo** | 48.81 |  |  | 48.92 |  |
| **AMT** | ***63.58*** |  |  |  |  |
| **AMT** | 59.51 | 10.71 | 1.11 | 59.36 | 10.48 |
| **AMT** | 59.26 | 10.46 | 1.12 | 59.16 | 10.27 |
| **AMT** | 58.75 | 9.95 | 1.11 | 58.49 | 9.6 |
| **Cpd-2** | 70.25 | 21.45 | 1.1 | 70.37 | 21.49 |
| **Cpd-2** | 70.45 | 21.65 | 1.41 | 70.39 | 21.51 |
| **Cpd-2** | 70.38 | 21.58 | 1.48 | 70.41 | 21.53 |
| **Cpd-2** | 69.96 | 21.17 | 1.78 | 70.08 | 21.2 |
| **Cpd-2 + AMT** | 73.98 | 25.18 | 1.18 | 74.55 | 25.67 |
| **Cpd-2 + AMT** | 73.7 | 24.9 | 1.16 | 74.22 | 25.34 |
| **Cpd-2 + AMT** | 73.6 | 24.8 | 1.15 | 73.99 | 25.1 |
| **Cpd-2 + AMT** | 73.33 | 24.53 | 1.14 | 73.66 | 24.77 |
| **Cpd-1** | 59.1 | 10.3 | 1.06 | 55.45 | 6.57 |
| **Cpd-1** | 60.21 | 11.41 | 1.06 | 56.16 | 7.28 |
| **Cpd-1** | 60.17 | 11.37 | 1.05 | 55.84 | 6.95 |
| **Cpd-1** | 60.23 | 11.43 | 1.06 | 55.85 | 6.97 |
| **Cpd-1 + AMT** | 70.89 | 22.09 | 1.13 | 71.43 | 22.55 |
| **Cpd-1 + AMT** | 70.91 | 22.12 | 1.13 | 71.79 | 22.91 |
| **Cpd-1 + AMT** | 70.25 | 21.45 | 1.18 | 71.21 | 22.33 |
| **Cpd-1 + AMT** | 69.66 | 20.86 | 1.2 | 71.22 | 22.34 |

**Supplementary Table S2.** Differential scanning fluorimetry data corresponding to supplementary figure S4. The experiments were carried out in quadruplicate, or in triplicate where single outlier values (highlighted in bold italics) were excluded from calculation of the melting curves. AMT was added at a concentration of 500 µM; Cpd-1 and Cpd-1 were added at a concentration of 25 µM.

**
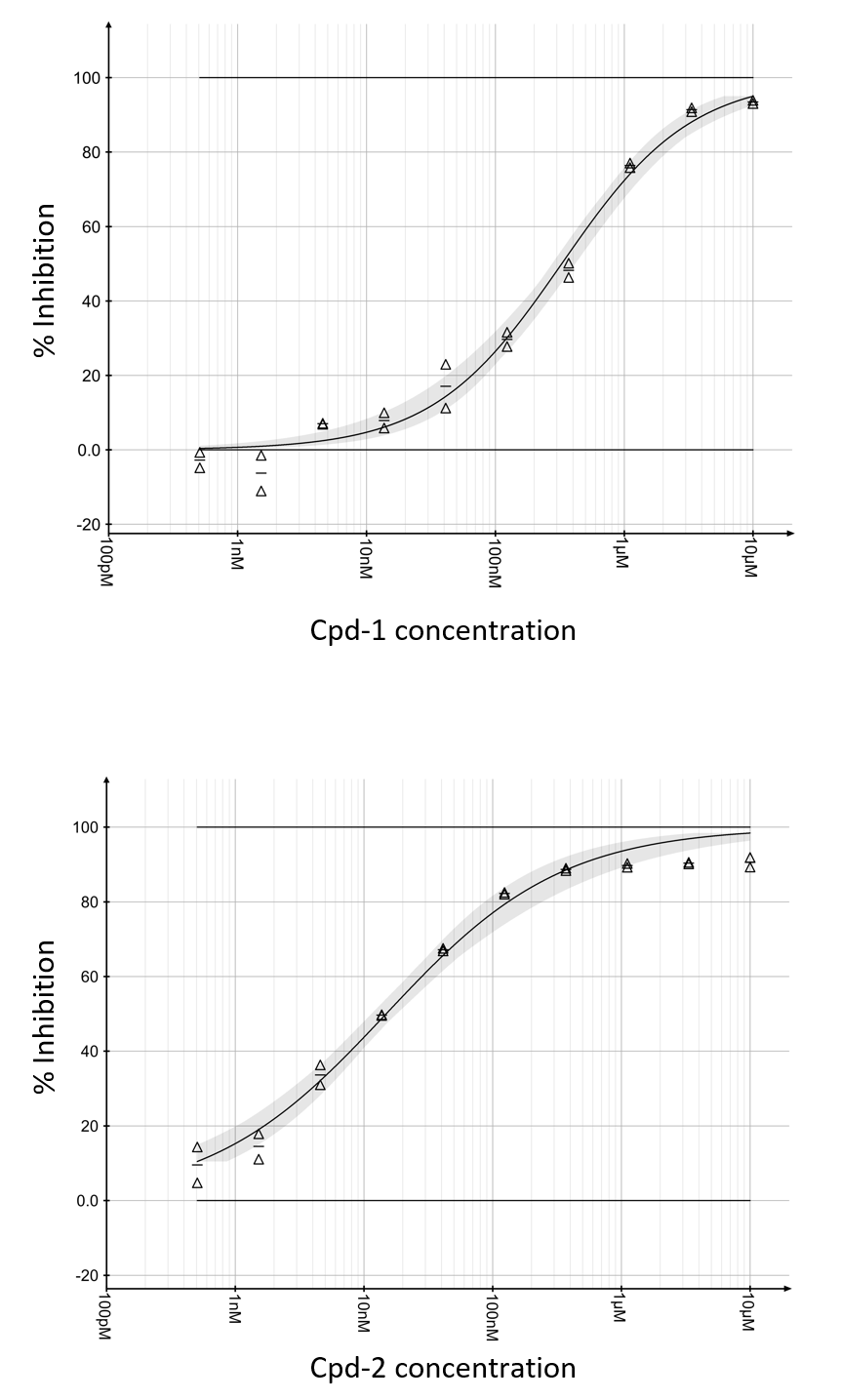
**

**Supplementary Figure S1.** IC_50_ determination of inhibitor Cpd-1 and Cpd-2 in the TDO2 SW48 cellular assay as described in the Methods section. SW48 cells were incubated for 24 hours with the inhibitor before NFK and KYN were quantified by LCMS. Percent inhibition of NFK and KYN production is plotted for each inhibitor.


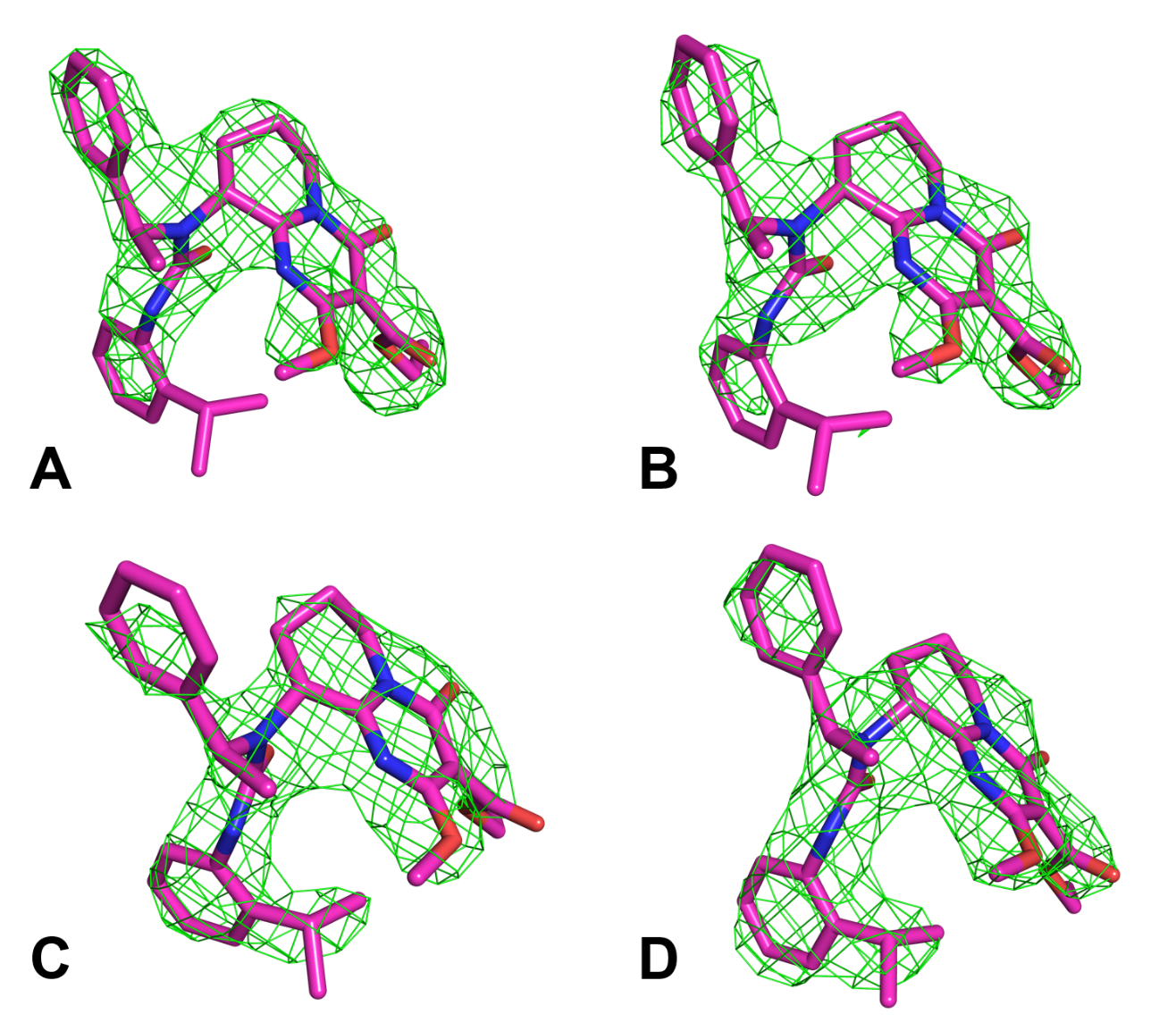


**Supplementary Figure S2.** Fo-Fc omit electron density maps (green, 2.61 Å resolution) contoured at 3 sigma level, showing Cpd-2 bound to TDO2 chains A-D (matching figure lettering). Cpd-2 is shown as sticks with atom color magenta (C), blue (N) and red (O). The structure was refined with (BUSTER refinement with 5 full cycles) in the absence of inhibitor before calculation of the omit map.


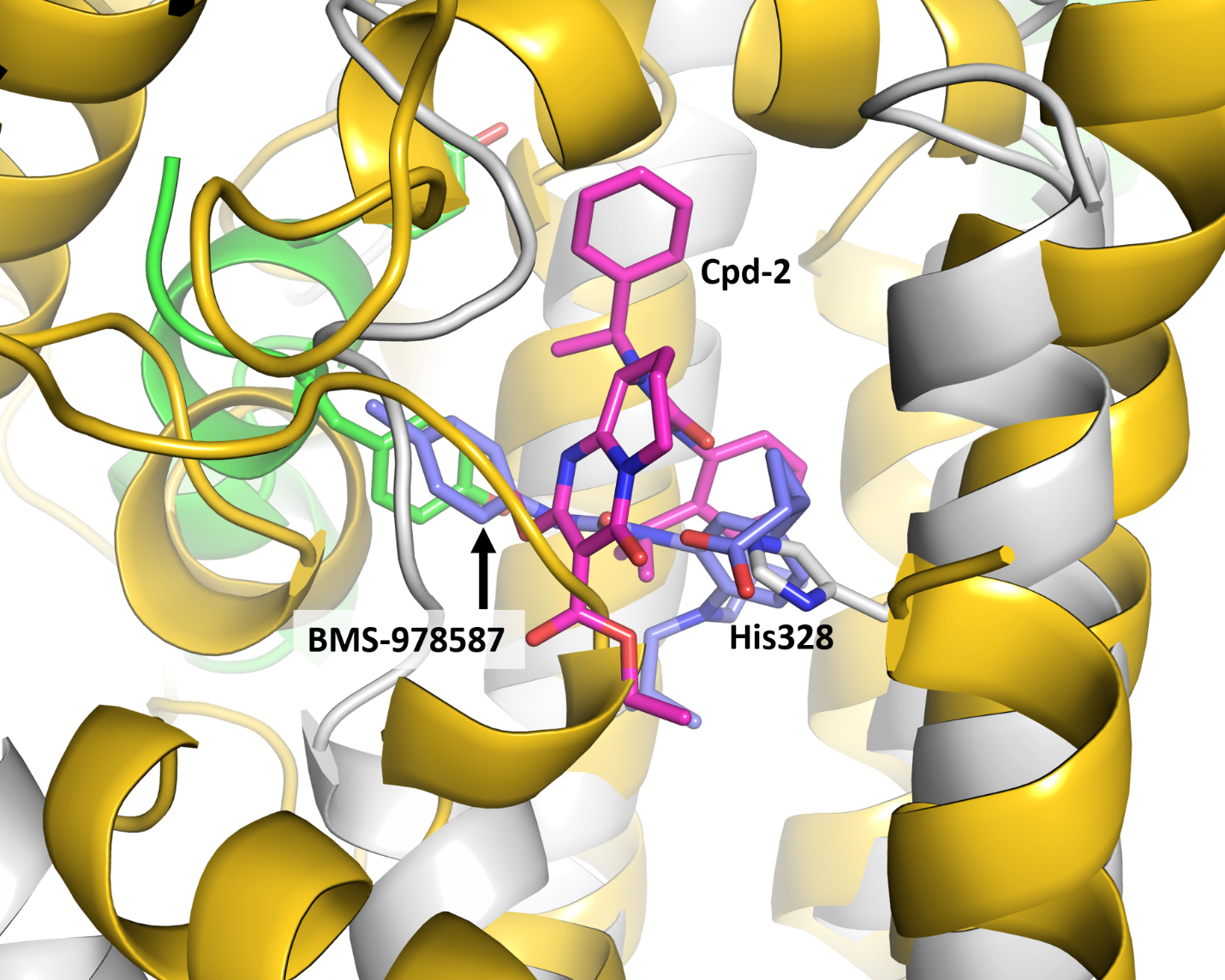


**Supplementary Figure S3.** A superposition of the Cpd-2 / apo-TDO2 inhibitor complex with the complex of apo-IDO1 and the inhibitor BMS-978587 (PDB ID 6AZV), showing the low structural similarity between the two enzymes, and the different chemical structures and binding modes of their inhibitors.

**
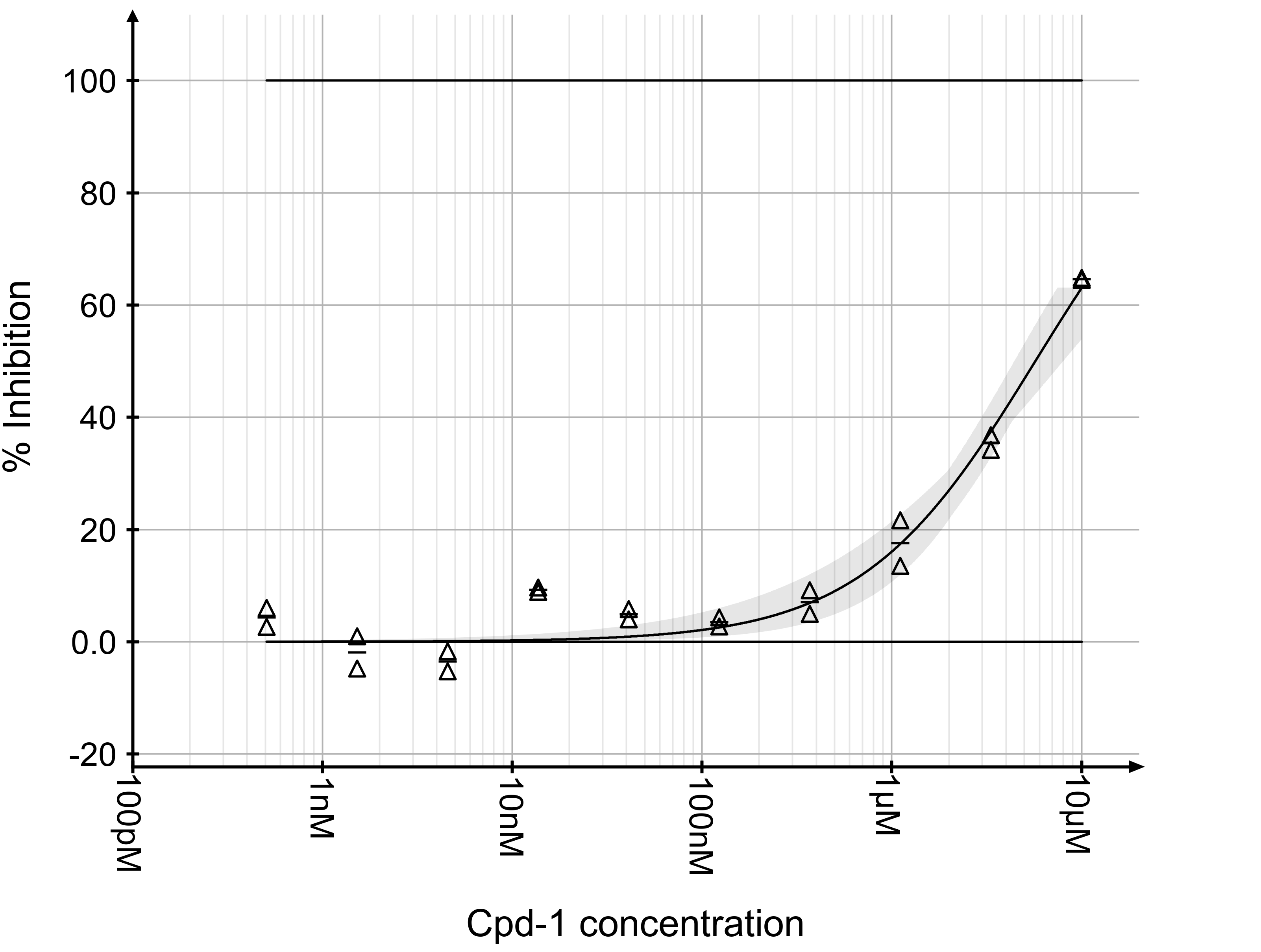
**

**
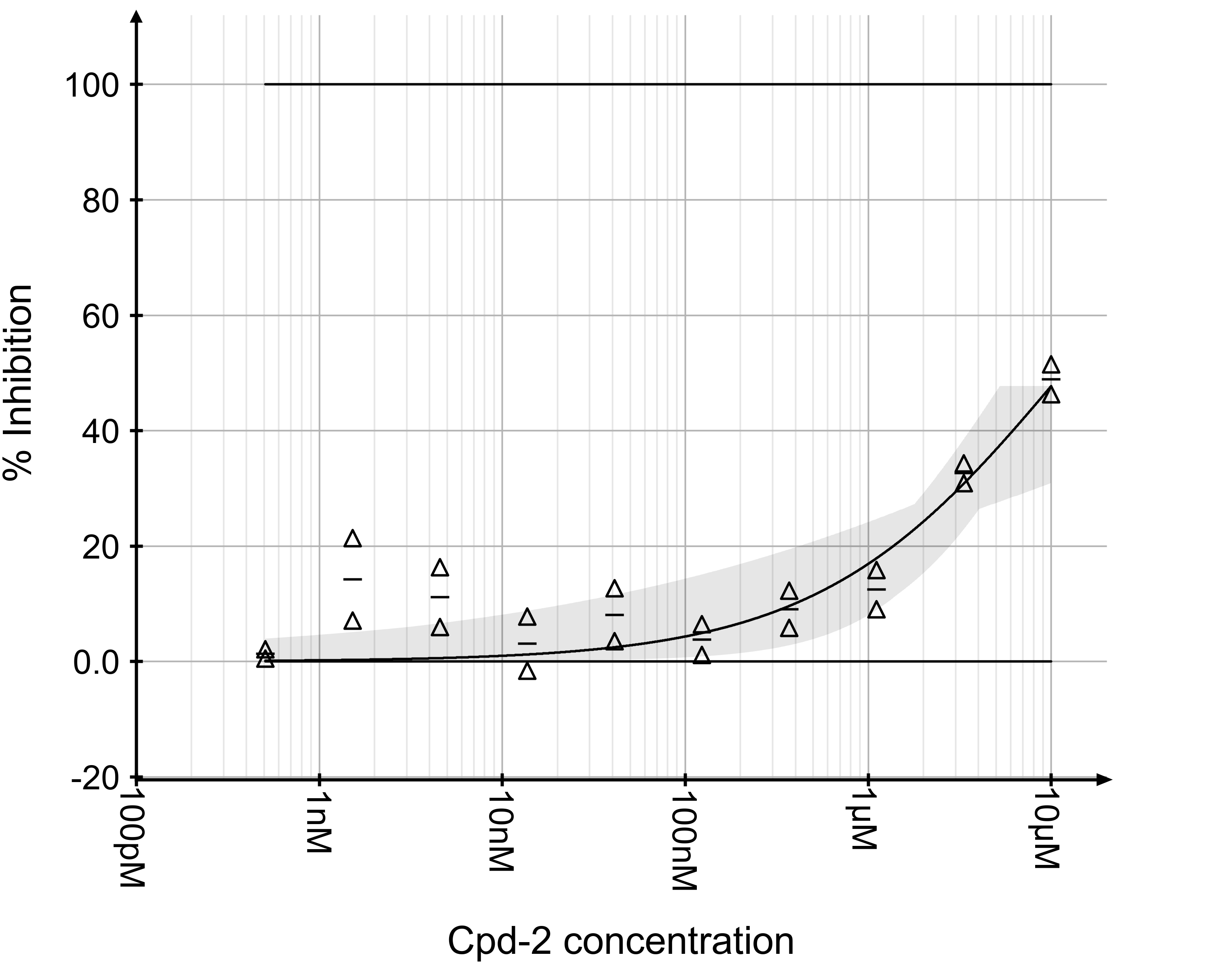
**

**Supplementary Figure S4.** IC_50_ determination of inhibitor Cpd-1 and Cpd-2 in the IDO1 SKOV3 cellular assay as described in the Methods section. SKOV3 cells treated with IFNγ to induce IDO expression were incubated for 24 hours with the inhibitor before NFK and KYN were quantified by LCMS. Percent inhibition of NFK and KYN production is plotted for each inhibitor.


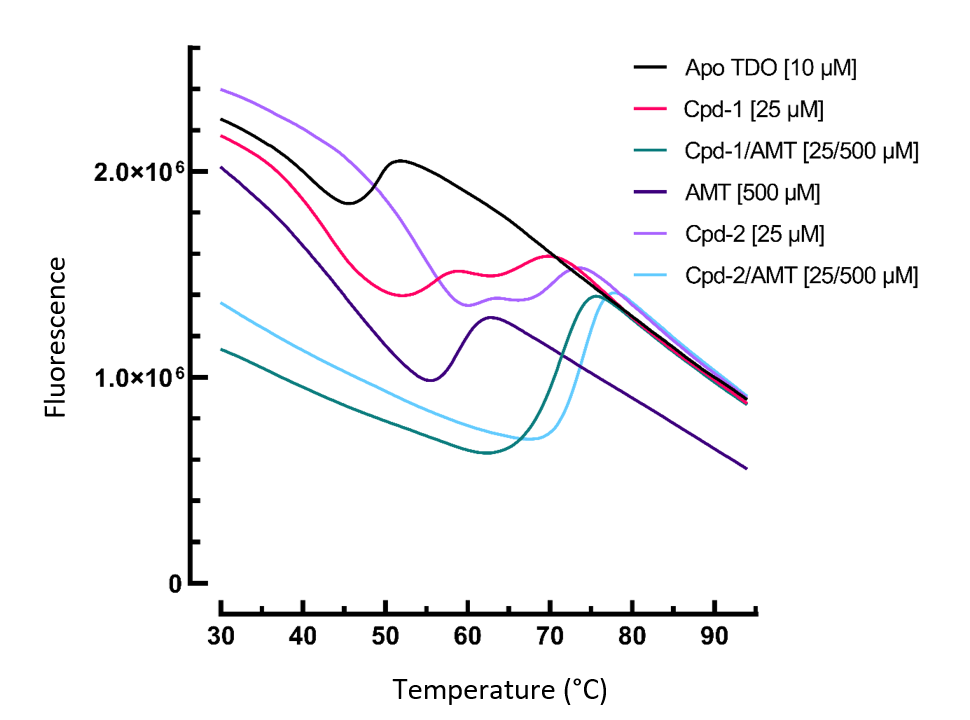


**Supplementary Figure S5.** The differential scanning fluorimetry melting curves of apo-TDO2 alone, in the presence of 500 µM AMT, and in the presence of 500 µM AMT and 25 µM Cpd-1 or Cpd-2.


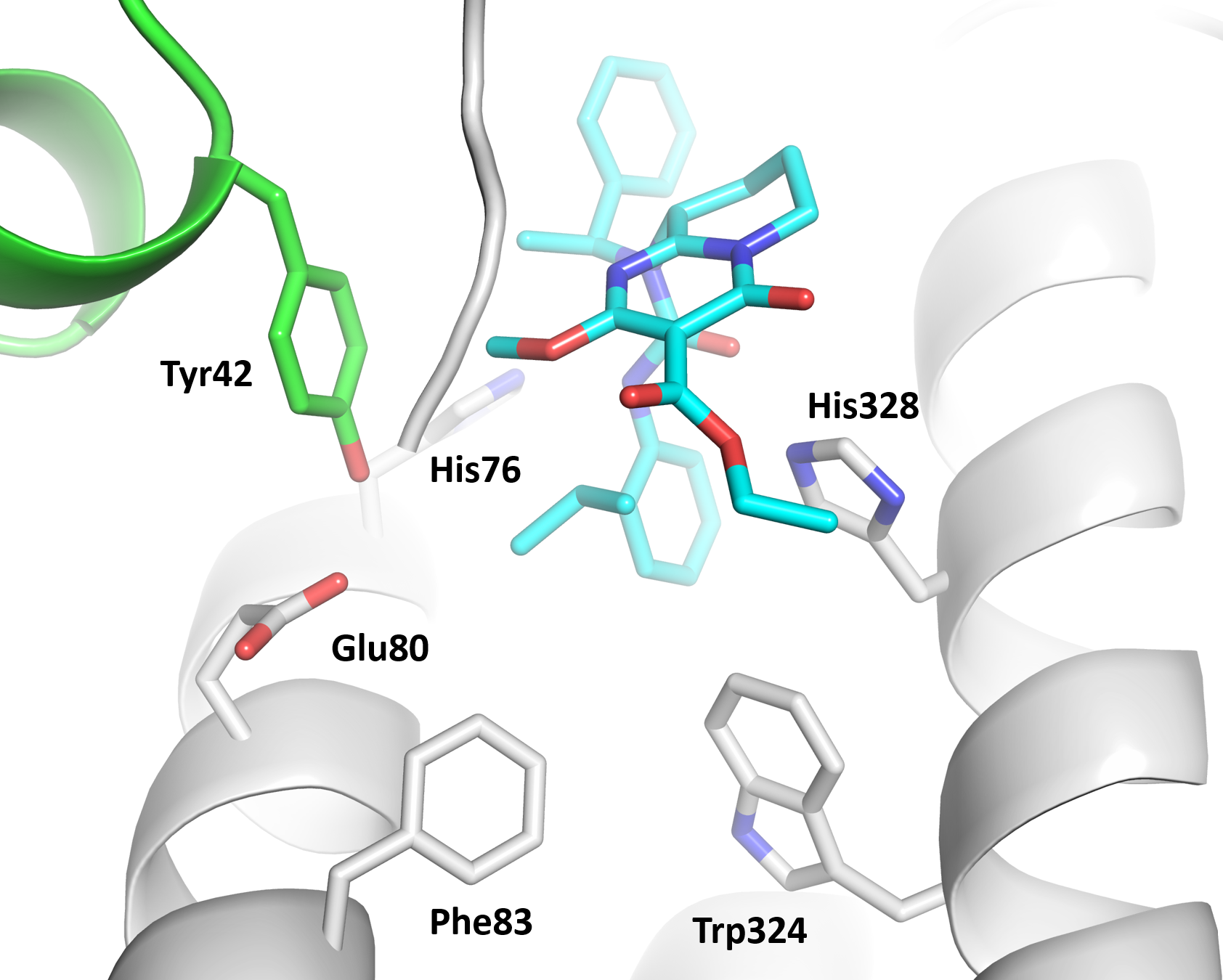


**Supplementary Figure S6.** The residues adjacent to the isopropyl group of Cpd-2 are shown as sticks in grey (chain A) or green (chain D). Residues 156-162 are omitted for clarity.

Supplementary Figure S7: The UV-VIS absorption spectrum of apo-TDO2.


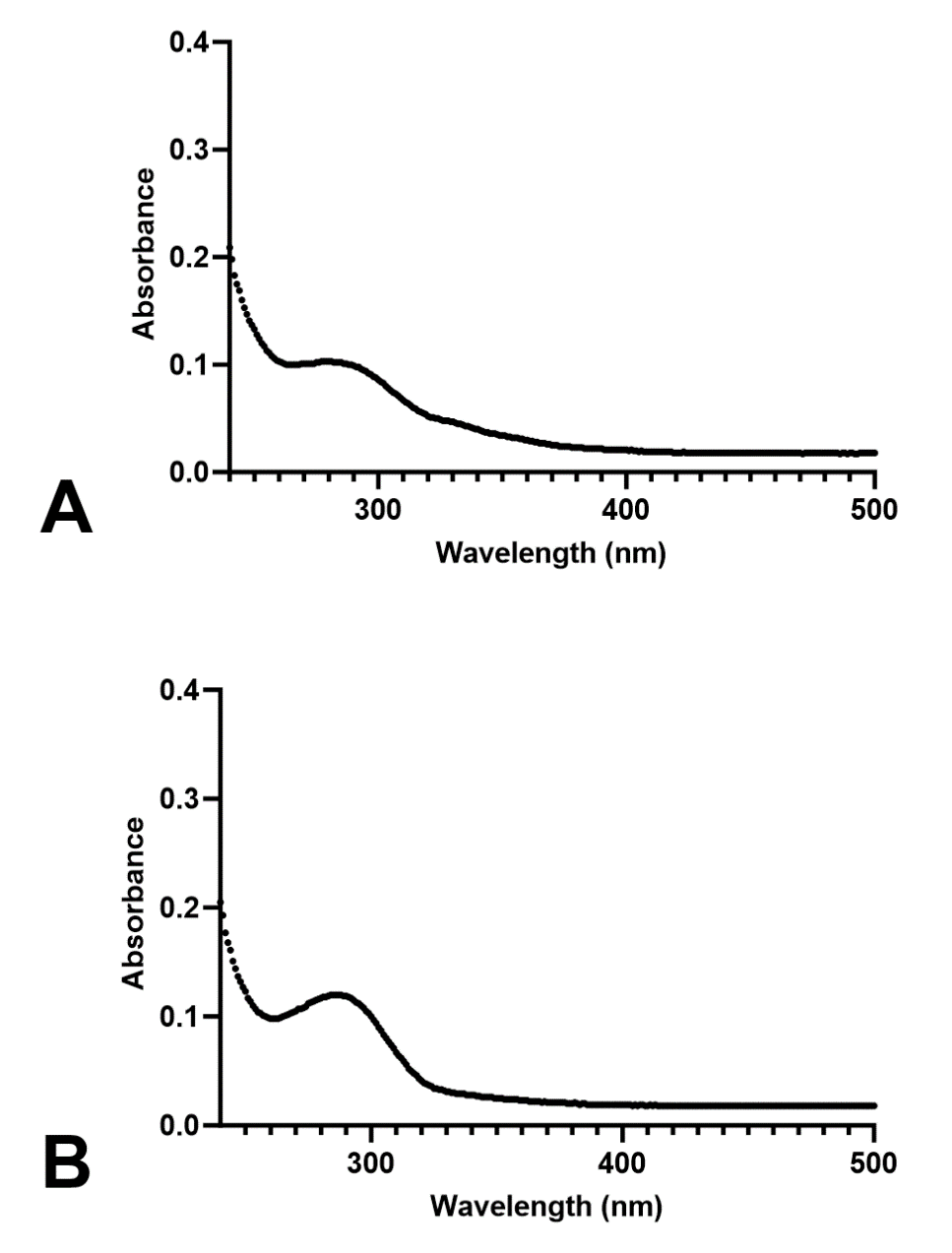


Supplementary Figure S8: The UV-VIS absorption spectra of Cpd-1 (A) and Cpd-2 (B).
